# Supplementary material for: Axon guidance genes modulate neurotoxicity of ALS-associated UBQLN2
Source: eLife. 2023 Apr 11;12:e84382. doi: 10.7554/eLife.84382 (PMC10147378; doi:10.7554/eLife.84382)
Supplement: Figure 1—figure supplement 1—source data 1. [file elife-84382-fig1-figsupp1-data1.zip › Figure 1-Figure supplement 1 (A,B)/Figure 1-Figure supplement 1-1 uncropped.pdf]

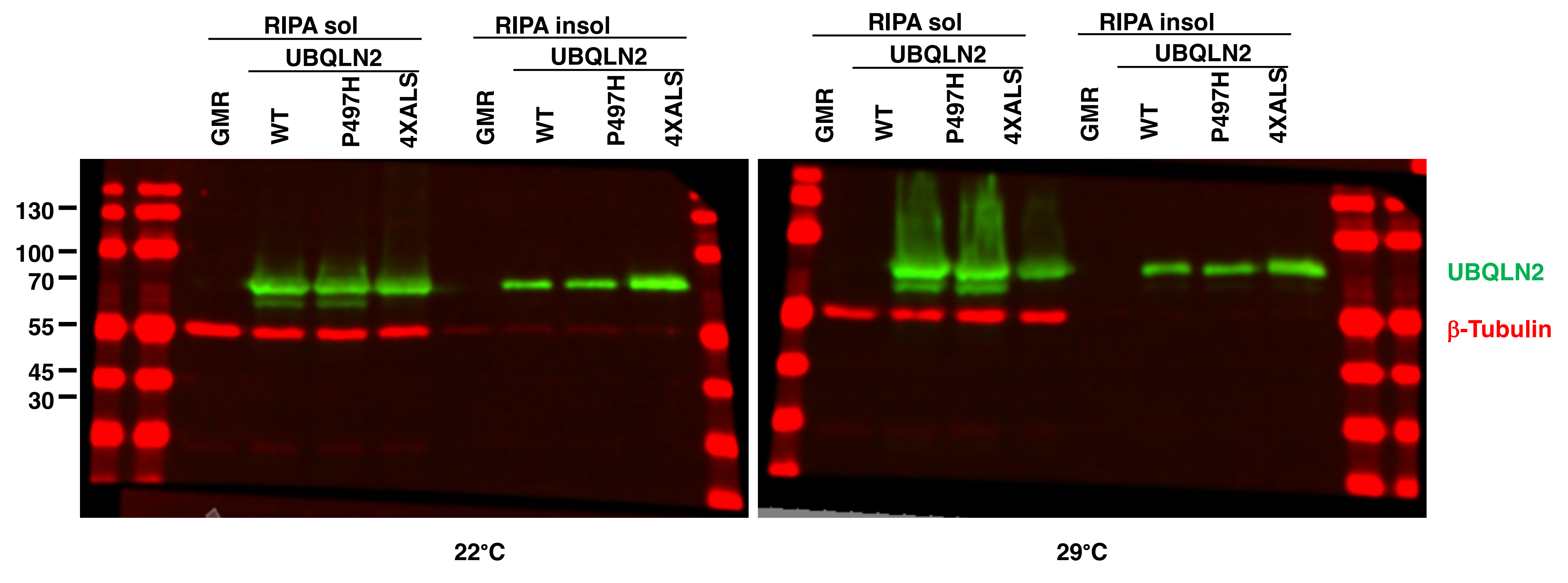
RIPA sol
UBQLN2
GMR
WT
P497H
4XALS
RIPA insol
UBQLN2
GMR
WT
P497H
4XALS
RIPA sol
UBQLN2
GMR
WT
P497H
4XALS
RIPA insol
UBQLN2
GMR
WT
P497H
4XALS
130
100
70
55
45
30
UBQLN2
β-Tubulin
22°C
29°C
