# Supplementary figures and images for: Axon guidance genes modulate neurotoxicity of ALS-associated UBQLN2
Source: eLife. 2023 Apr 11;12:e84382. doi: 10.7554/eLife.84382 (PMC10147378; doi:10.7554/eLife.84382)

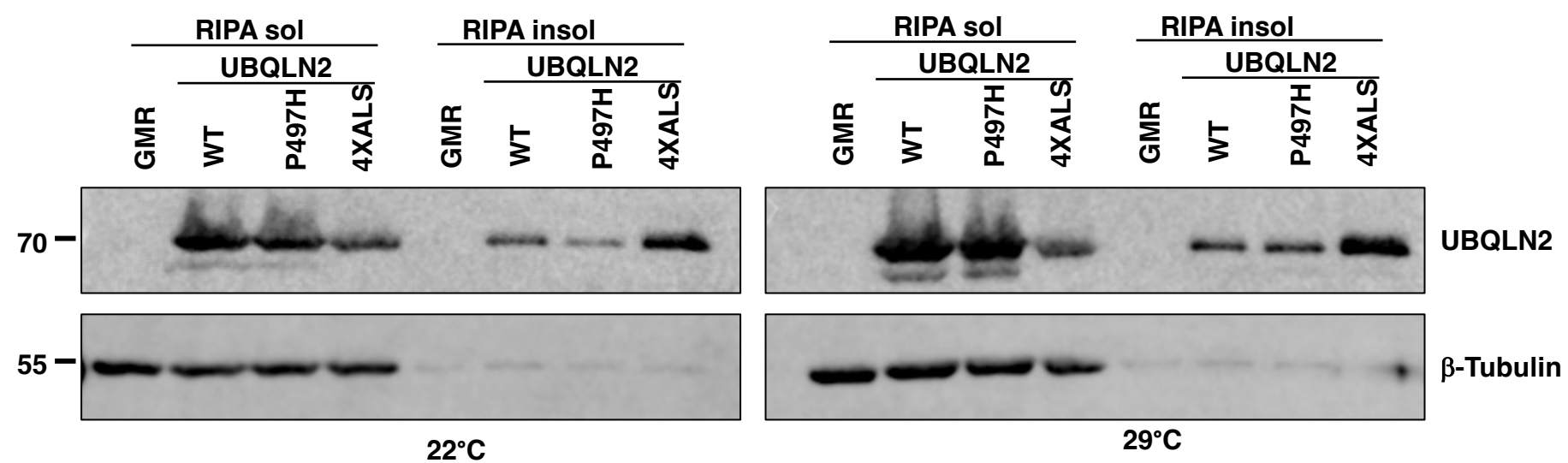

Supplement: Figure 1—figure supplement 1—source data 1. [file elife-84382-fig1-figsupp1-data1.zip › Figure 1-Figure supplement 1 (A,B)/Figure 1-Figure supplement 1-3.pdf]

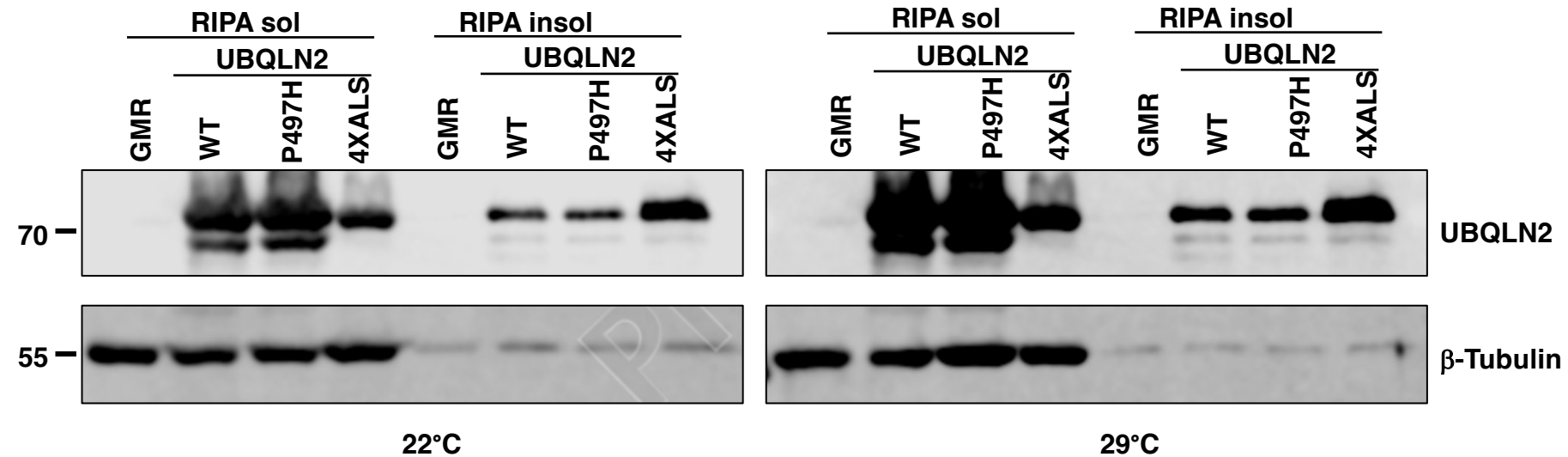

Supplement: Figure 1—figure supplement 1—source data 1. [file elife-84382-fig1-figsupp1-data1.zip › Figure 1-Figure supplement 1 (A,B)/Figure 1-Figure supplement 1-2.pdf]

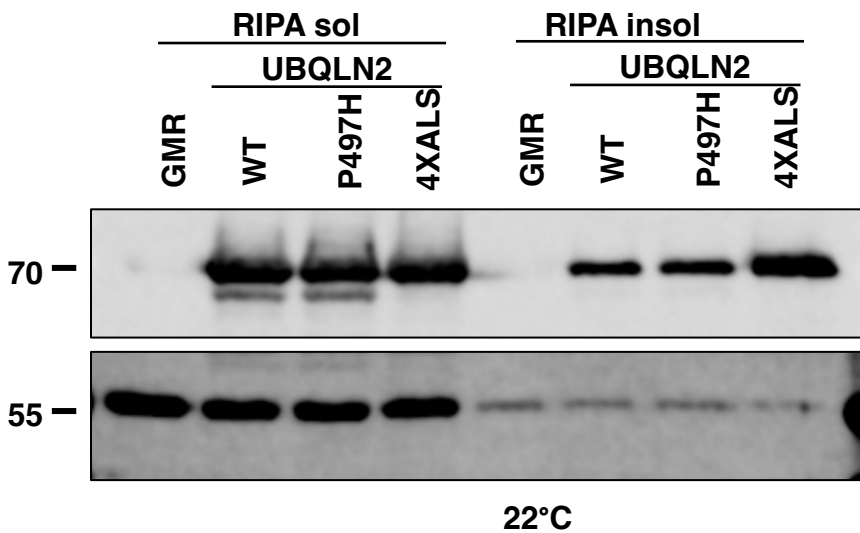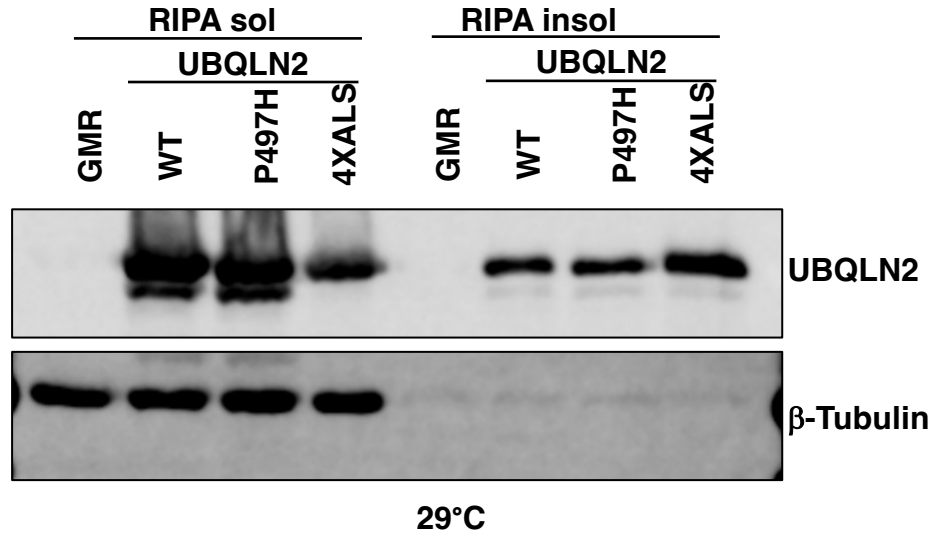

Supplement: Figure 1—figure supplement 1—source data 1. [file elife-84382-fig1-figsupp1-data1.zip › Figure 1-Figure supplement 1 (A,B)/Figure 1-Figure supplement 1-1.pdf]

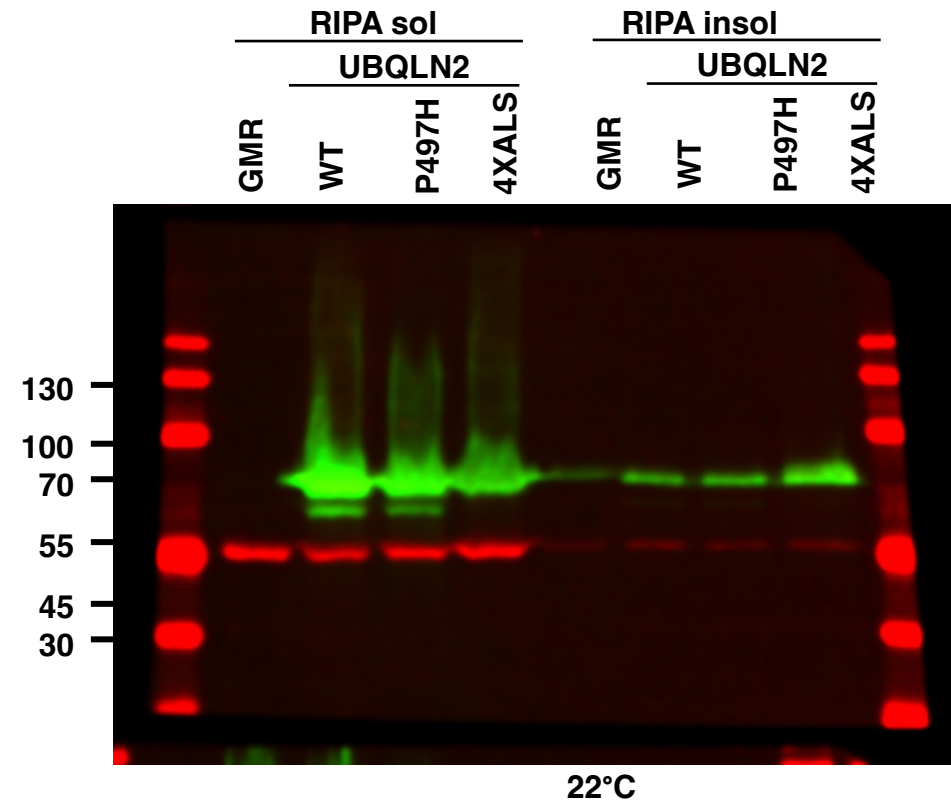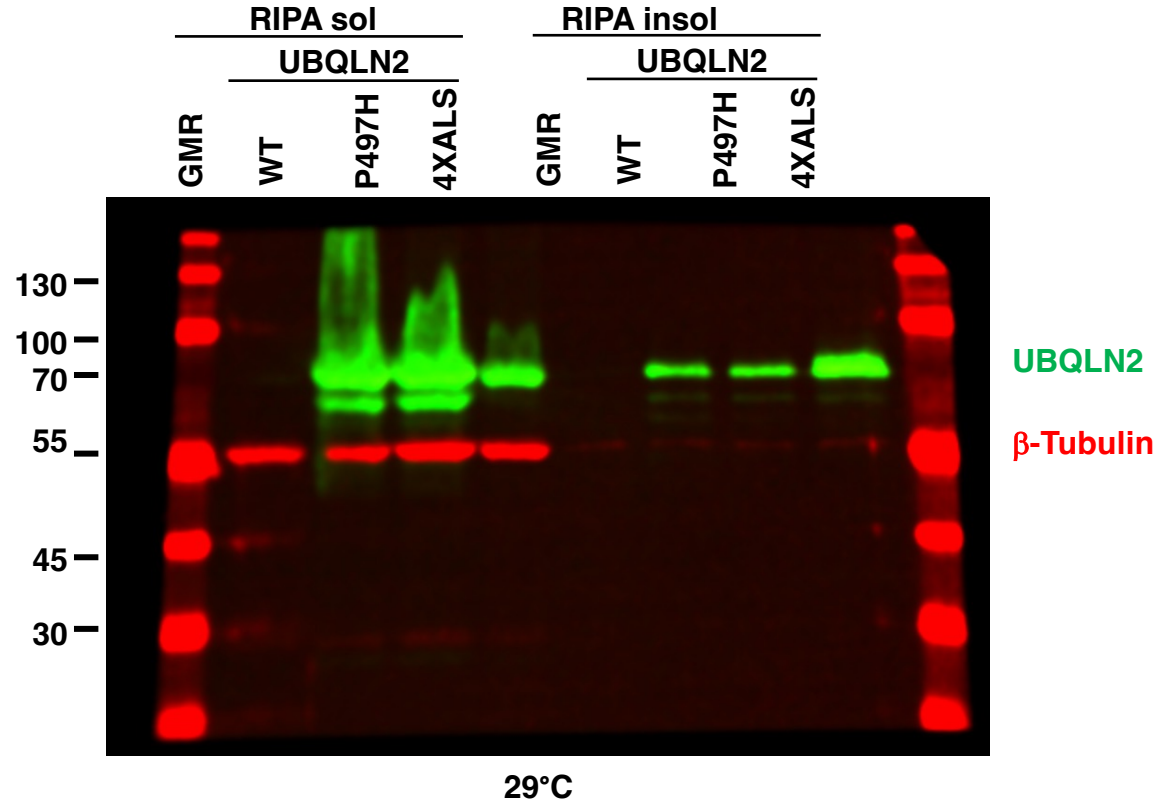

Supplement: Figure 1—figure supplement 1—source data 1. [file elife-84382-fig1-figsupp1-data1.zip › Figure 1-Figure supplement 1 (A,B)/Figure 1-Figure supplement 1-2 uncropped.pdf]

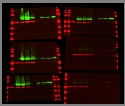

Supplement: Figure 1—figure supplement 1—source data 1. [file elife-84382-fig1-figsupp1-data1.zip › Figure 1-Figure supplement 1 (A,B)/Figure 1-Figure supplement 1-original/0004505_08_TH.jpg]

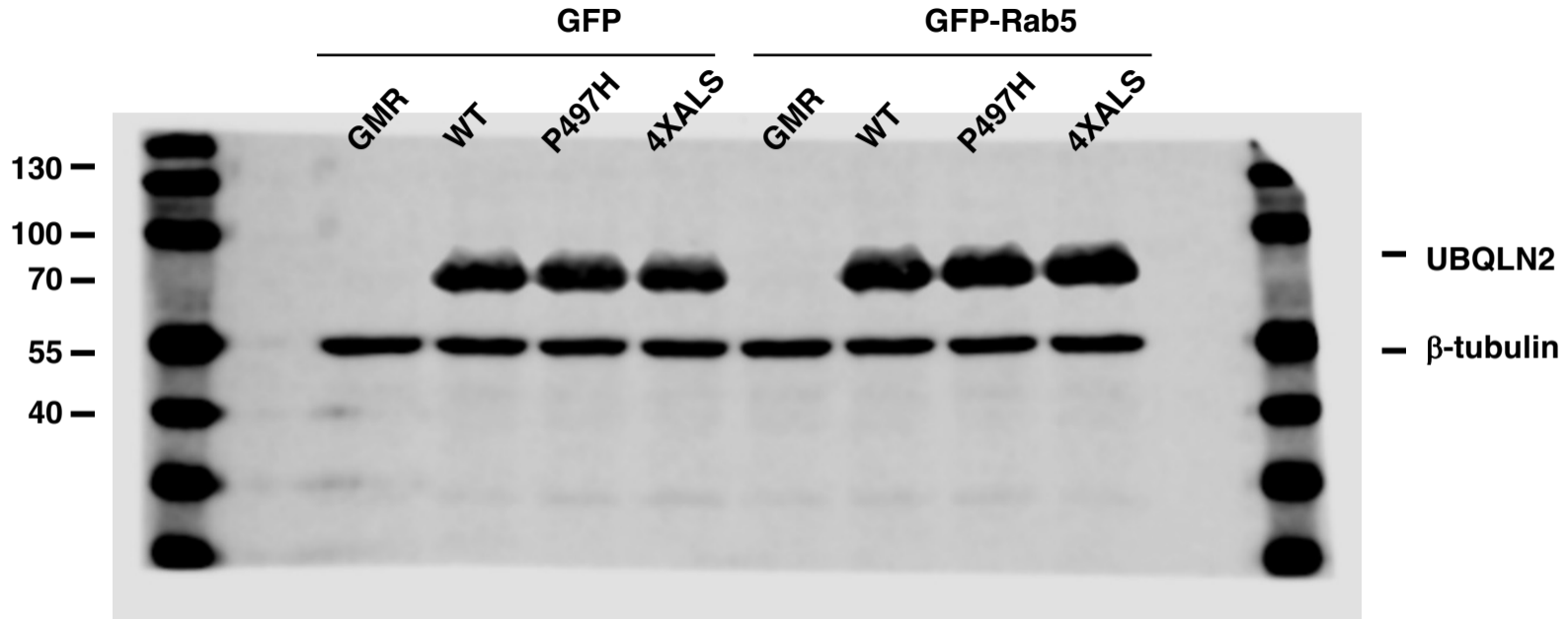

Supplement: Figure 2—figure supplement 1—source data 1. [file elife-84382-fig2-figsupp1-data1.zip › Figure 2-Figure supplement 1 source data/Figure 2-Figure supplement 1 (G,H)/Figure 2-Figure supplement 1 (G,H)-2 uncropped.pdf]

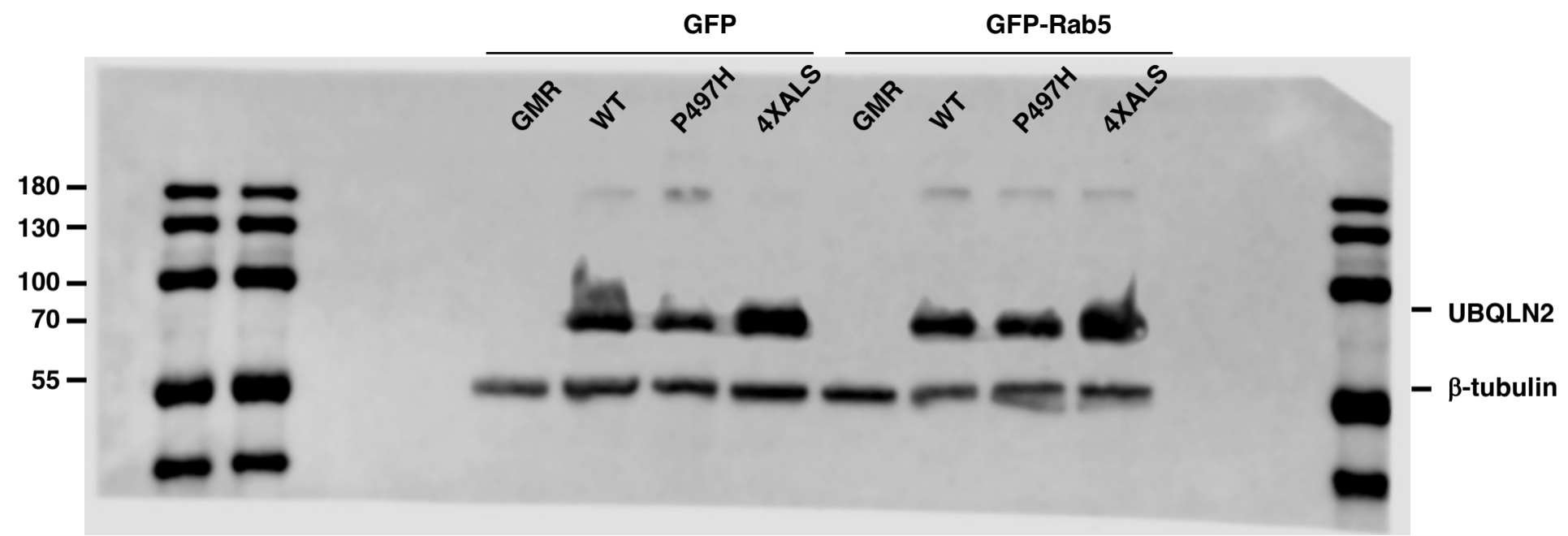

Supplement: Figure 2—figure supplement 1—source data 1. [file elife-84382-fig2-figsupp1-data1.zip › Figure 2-Figure supplement 1 source data/Figure 2-Figure supplement 1 (G,H)/Figure 2-Figure supplement 1 (G,H)-1 uncropped.pdf]

**GFP**

**GFP-Rab5**

**GMR**

**WT**

**P497H**

**4XALS**

**GMR**

**WT**

**P497H**

**4XALS**

70

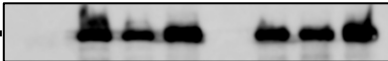

**UBQLN2**

55

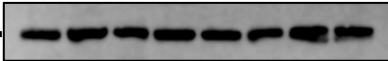

**$\beta$ -tubulin**

Supplement: Figure 2—figure supplement 1—source data 1. [file elife-84382-fig2-figsupp1-data1.zip › Figure 2-Figure supplement 1 source data/Figure 2-Figure supplement 1 (G,H)/Figure 2-Figure supplement 1 (G,H)-1.pdf]

**GFP**

**GFP-Rab5**

**GMR**

**WT**

**P497H**

**4XALS**

**GMR**

**WT**

**P497H**

**4XALS**

70

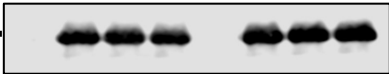

**UBQLN2**

55

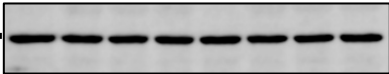

**$\beta$ -tubulin**

Supplement: Figure 2—figure supplement 1—source data 1. [file elife-84382-fig2-figsupp1-data1.zip › Figure 2-Figure supplement 1 source data/Figure 2-Figure supplement 1 (G,H)/Figure 2-Figure supplement 1 (G,H)-2.pdf]

**GFP**

**GFP-Rab5**

**GMR**

**WT**

**P497H**

**4XALS**

**GMR**

**WT**

**P497H**

**4XALS**

70

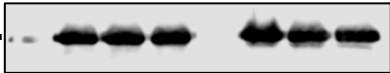

**UBQLN2**

55

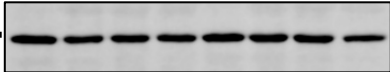

**$\beta$ -tubulin**

Supplement: Figure 2—figure supplement 1—source data 1. [file elife-84382-fig2-figsupp1-data1.zip › Figure 2-Figure supplement 1 source data/Figure 2-Figure supplement 1 (G,H)/Figure 2-Figure supplement 1 (G,H)-3.pdf]

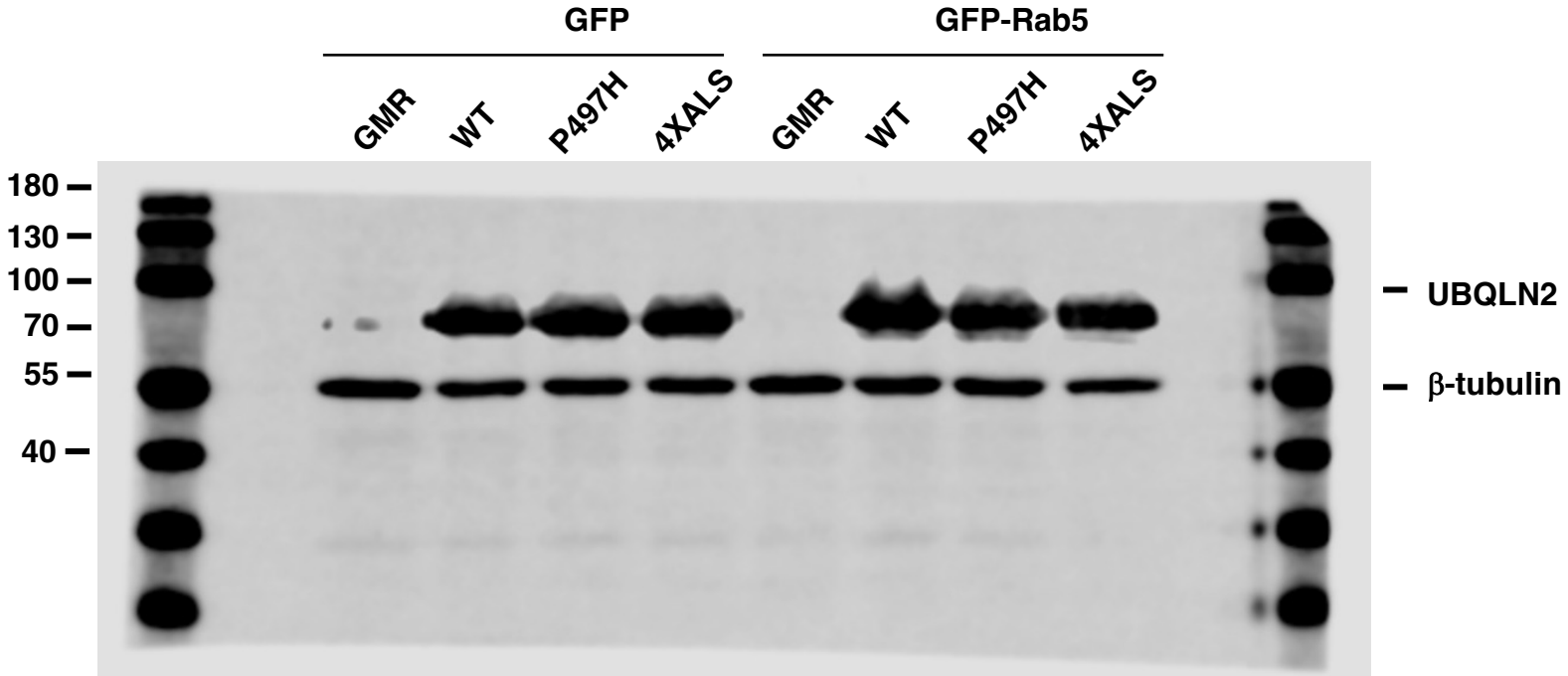

Supplement: Figure 2—figure supplement 1—source data 1. [file elife-84382-fig2-figsupp1-data1.zip › Figure 2-Figure supplement 1 source data/Figure 2-Figure supplement 1 (G,H)/Figure 2-Figure supplement 1 (G,H)-3 uncropped.pdf]

shLuci

shRab5

GMR

WT

P497H

4XALS

GMR

WT

P497H

4XALS

UBQLN2

 $\beta$ -tubulin

— 180

— 130

— 100

— 70

— 55

— 40

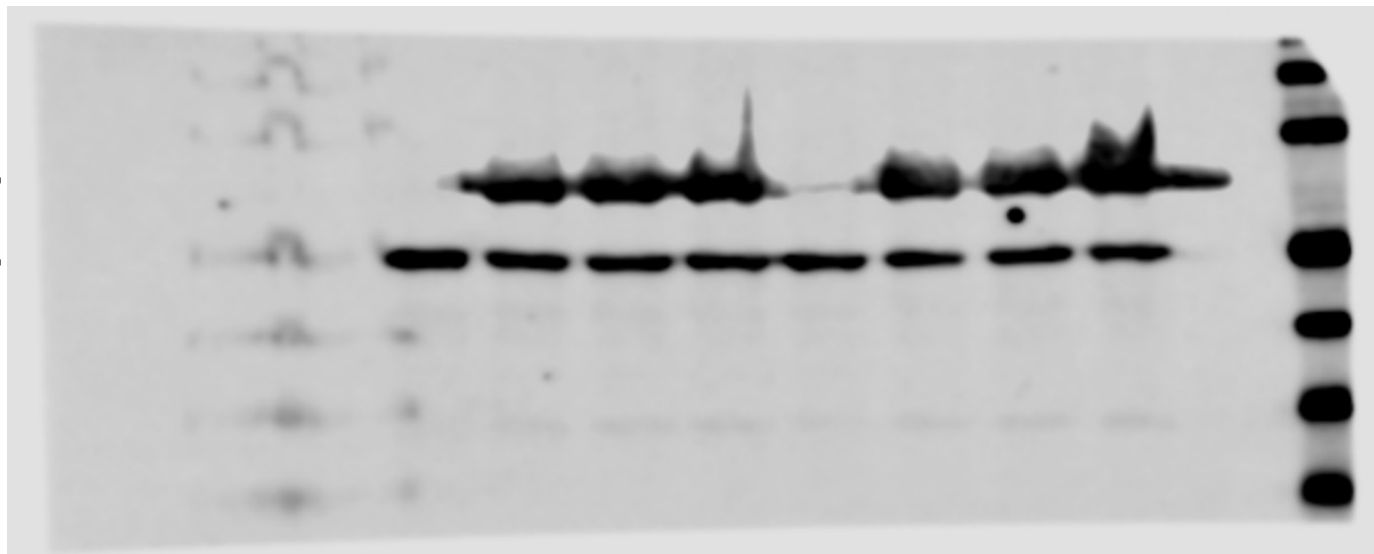

Supplement: Figure 2—figure supplement 1—source data 1. [file elife-84382-fig2-figsupp1-data1.zip › Figure 2-Figure supplement 1 source data/Figure 2-Figure supplement 1 (D,E)/Figure 2-Figure supplement 1 (D,E)-3 uncropped.pdf]

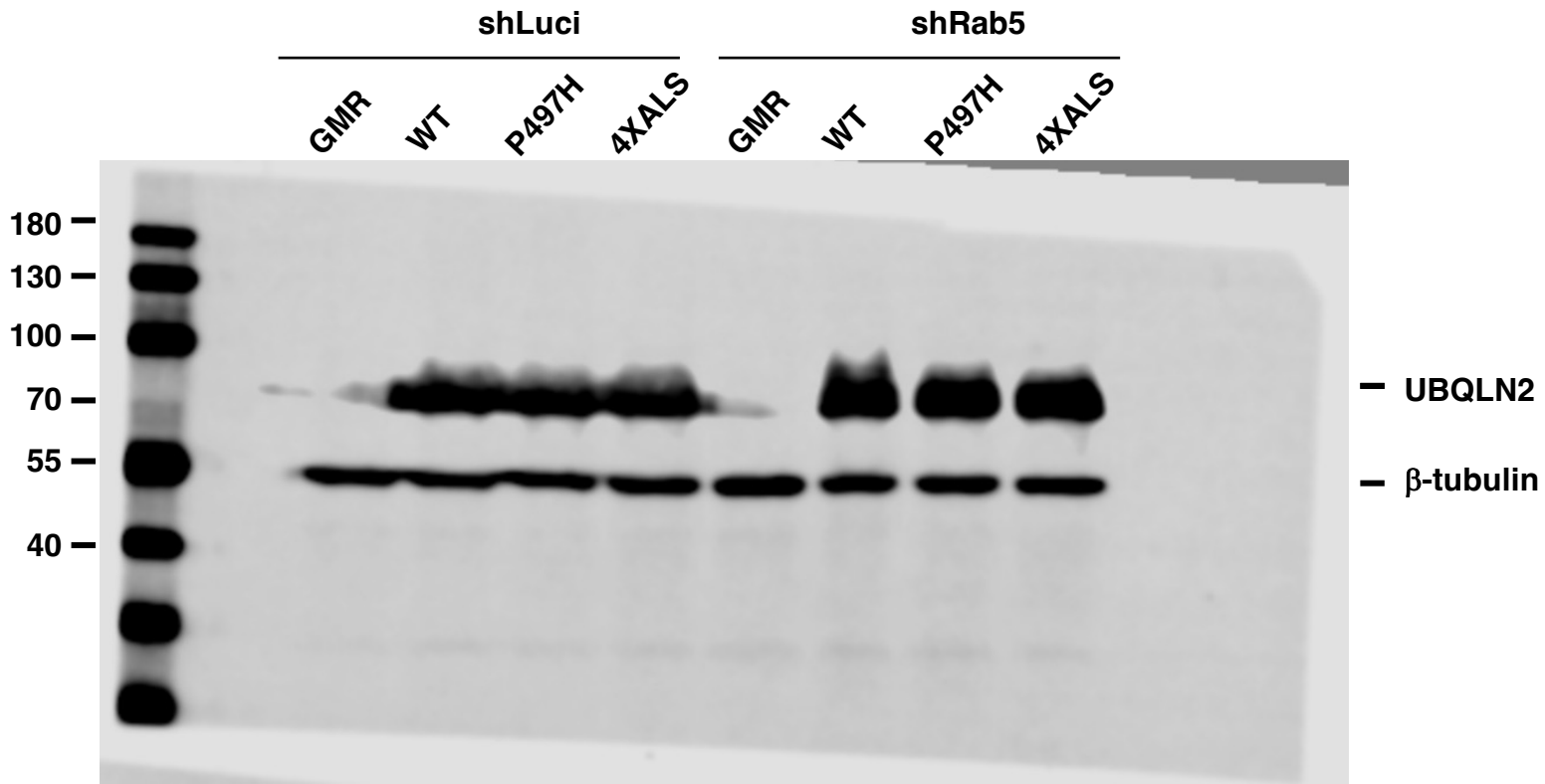

Supplement: Figure 2—figure supplement 1—source data 1. [file elife-84382-fig2-figsupp1-data1.zip › Figure 2-Figure supplement 1 source data/Figure 2-Figure supplement 1 (D,E)/Figure 2-Figure supplement 1 (D,E)-2 uncropped.pdf]

**shLuci**

**shRab5**

**GMR** **WT** **P497H** **4XALS** **GMR** **WT** **P497H** **4XALS**

70

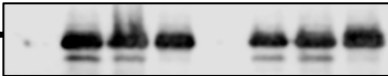

**UBQLN2**

55

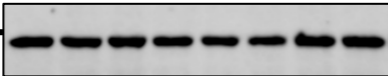

**$\beta$ -tubulin**

Supplement: Figure 2—figure supplement 1—source data 1. [file elife-84382-fig2-figsupp1-data1.zip › Figure 2-Figure supplement 1 source data/Figure 2-Figure supplement 1 (D,E)/Figure 2-Figure supplement 1 (D,E)-1.pdf]

**shLuci**

**shRab5**

**GMR**

**WT**

**P497H**

**4XALS**

**GMR**

**WT**

**P497H**

**4XALS**

70

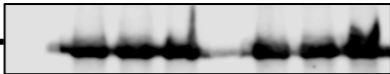

**UBQLN2**

55

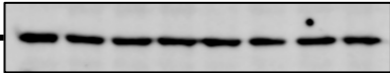

**$\beta$ -tubulin**

Supplement: Figure 2—figure supplement 1—source data 1. [file elife-84382-fig2-figsupp1-data1.zip › Figure 2-Figure supplement 1 source data/Figure 2-Figure supplement 1 (D,E)/Figure 2-Figure supplement 1 (D,E)-3.pdf]

**shLuci**

**shRab5**

**GMR**

**WT**

**P497H**

**4XALS**

**GMR**

**WT**

**P497H**

**4XALS**

70

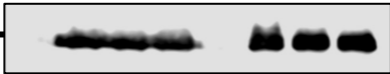

**UBQLN2**

55

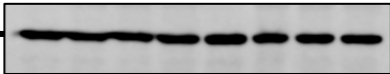

**$\beta$ -tubulin**

Supplement: Figure 2—figure supplement 1—source data 1. [file elife-84382-fig2-figsupp1-data1.zip › Figure 2-Figure supplement 1 source data/Figure 2-Figure supplement 1 (D,E)/Figure 2-Figure supplement 1 (D,E)-2.pdf]

shLuci

shRab5

GMR

WT

P497H

4XALS

GMR

WT

P497H

4XALS

UBQLN2

$\beta$ -tubulin

180

130

100

70

55

40

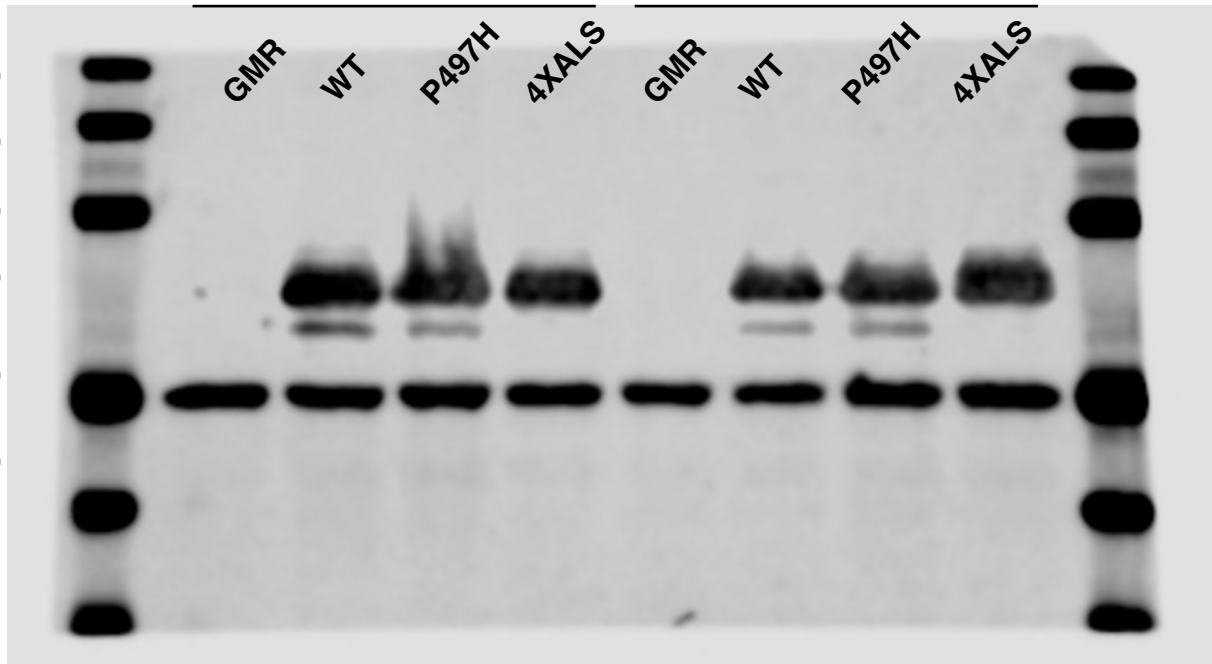

Supplement: Figure 2—figure supplement 1—source data 1. [file elife-84382-fig2-figsupp1-data1.zip › Figure 2-Figure supplement 1 source data/Figure 2-Figure supplement 1 (D,E)/Figure 2-Figure supplement 1 (D,E)-1 uncropped.pdf]

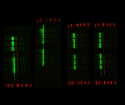

Supplement: Figure 2—figure supplement 1—source data 1. [file elife-84382-fig2-figsupp1-data1.zip › Figure 2-Figure supplement 1 source data/Figure 2-Figure supplement 1 (G,H)/Figure 2-Figure supplement 1 (G,H)-2,3-original/0004517_01_TH.jpg]

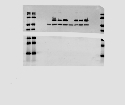

Supplement: Figure 2—figure supplement 1—source data 1. [file elife-84382-fig2-figsupp1-data1.zip › Figure 2-Figure supplement 1 source data/Figure 2-Figure supplement 1 (G,H)/Figure 2-Fifure supplement 1 (G,H)-1-original/0003134_01_TH.jpg]

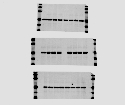

Supplement: Figure 2—figure supplement 1—source data 1. [file elife-84382-fig2-figsupp1-data1.zip › Figure 2-Figure supplement 1 source data/Figure 2-Figure supplement 1 (D,E)/Figure 2-Figure supplement 1 (D,E)-1-original/0004533_01_TH.jpg]

**GMR>UBQLN2<sup>4XALS</sup>/**

---

*lilli*<sup>17-2</sup>

**CyO**

**70**

—

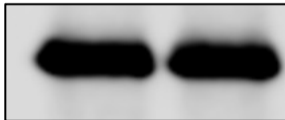

**UBQLN2**

**55**

—

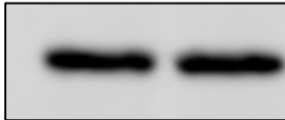

**$\beta$ -tubulin**

Supplement: Figure 2—figure supplement 2—source data 1. [file elife-84382-fig2-figsupp2-data1.zip › Figure 2-Figure supplement 2 source data/Figure 2-Figure supplement 2 (C)-1.pdf]

**GMR>UBQLN2<sup>4XALS</sup>/**

*lilli*<sup>17-2</sup>

**CyO**

70 —

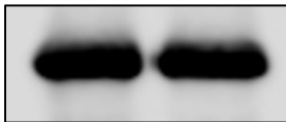

**UBQLN2**

55 —

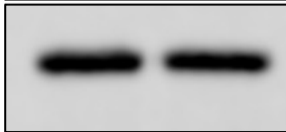

**$\beta$ -tubulin**

Supplement: Figure 2—figure supplement 2—source data 1. [file elife-84382-fig2-figsupp2-data1.zip › Figure 2-Figure supplement 2 source data/Figure 2-Figure supplement 2 (C)-2.pdf]

**GMR>UBQLN2<sup>4XALS</sup>/**

*lilli*<sup>17-2</sup>

**CyO**

**70**

—

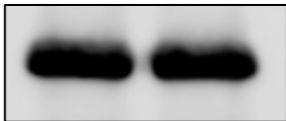

**UBQLN2**

**55**

—

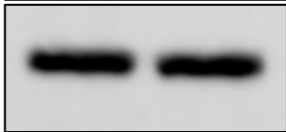

**$\beta$ -tubulin**

Supplement: Figure 2—figure supplement 2—source data 1. [file elife-84382-fig2-figsupp2-data1.zip › Figure 2-Figure supplement 2 source data/Figure 2-Figure supplement 2 (C)-3.pdf]

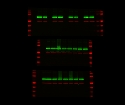

Supplement: Figure 2—figure supplement 2—source data 1. [file elife-84382-fig2-figsupp2-data1.zip › Figure 2-Figure supplement 2 source data/Figure 2-Figure supplement 2 (C)-original/0004534_01_TH.jpg]

GMR>UBQLN2<sup>4XALS</sup>

ED2426

CyO

BSC346

CyO

CyO

Unc-5<sup>3</sup>

shLuci

shUnc-5

130 —

100 —

70 —

55 —

45 —

30 —

UBQLN2

β-tubulin

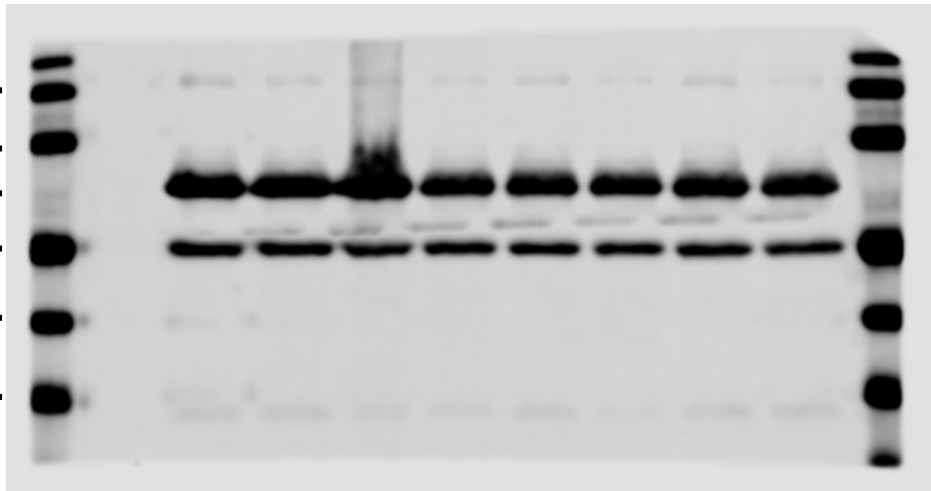

Supplement: Figure 3—source data 1. [file elife-84382-fig3-data1.zip › Figure 3 (D,E) source data/Figure 3 (D,E)-3 uncropped.pdf]

**GMR>UBQLN2<sup>4XALS</sup>**

ED2426

CyO

BSC346

CyO

CyO

Unc-5<sup>3</sup>

shLuci

shUnc-5

70

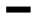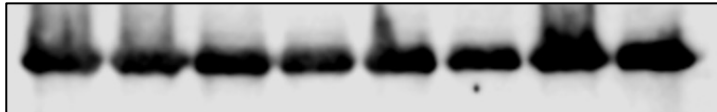

**UBQLN2**

55

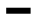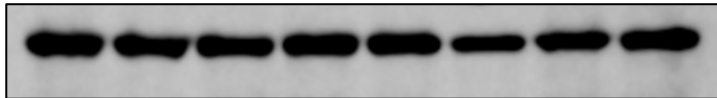

**β-tubulin**

Supplement: Figure 3—source data 1. [file elife-84382-fig3-data1.zip › Figure 3 (D,E) source data/Figure 3 (D,E)-2.pdf]

**GMR>UBQLN2<sup>4XALS</sup>**

**ED2426**

**CyO**

**BSC346**

**CyO**

**CyO**

**Unc-5<sup>3</sup>**

**shLuci**

**shUnc-5**

**70**

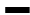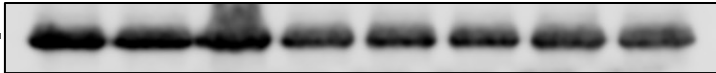

**UBQLN2**

**55**

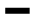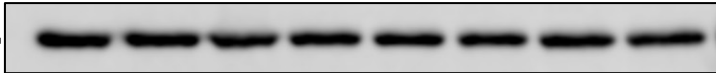

**β-tubulin**

Supplement: Figure 3—source data 1. [file elife-84382-fig3-data1.zip › Figure 3 (D,E) source data/Figure 3 (D,E)-3.pdf]

GMR>UBQLN2<sup>4XALS</sup>

ED2426

CyO

BSC346

CyO

CyO

Unc-53

shLuci

shUnc-5

130 —

100 —

70 —

55 —

45 —

30 —

UBQLN2

β-tubulin

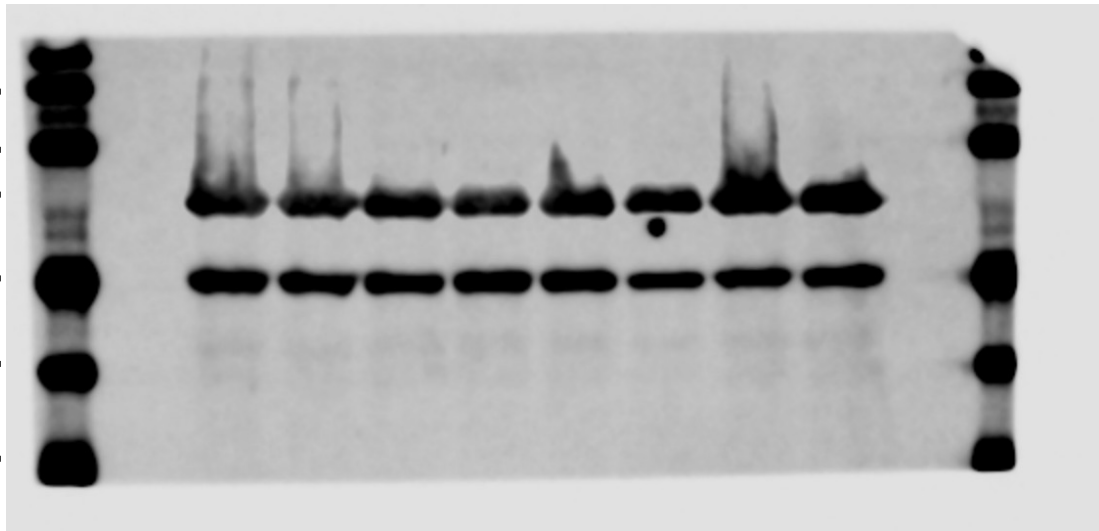

Supplement: Figure 3—source data 1. [file elife-84382-fig3-data1.zip › Figure 3 (D,E) source data/Figure 3 (D,E)-2 uncropped.pdf]

# GMR>UBQLN2<sup>4XALS</sup>

ED2426

CyO

BSC346

CyO

CyO

Unc-5<sup>3</sup>

shLuci

shUnc-5

70

—

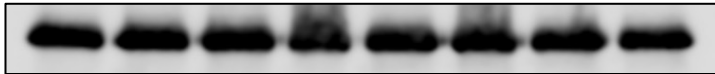

UBQLN2

55

—

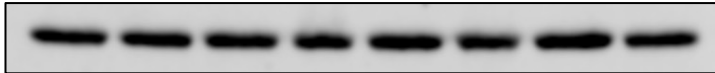

$\beta$ -tubulin

Supplement: Figure 3—source data 1. [file elife-84382-fig3-data1.zip › Figure 3 (D,E) source data/Figure 3 (D,E)-1.pdf]

**GMR>UBQLN2<sup>4XALS</sup>**

ED2426

CyO

BSC346

CyO

CyO

Unc-5<sup>3</sup>

shLuci

shUnc-5

130

100

70

55

45

30

UBQLN2

β-tubulin

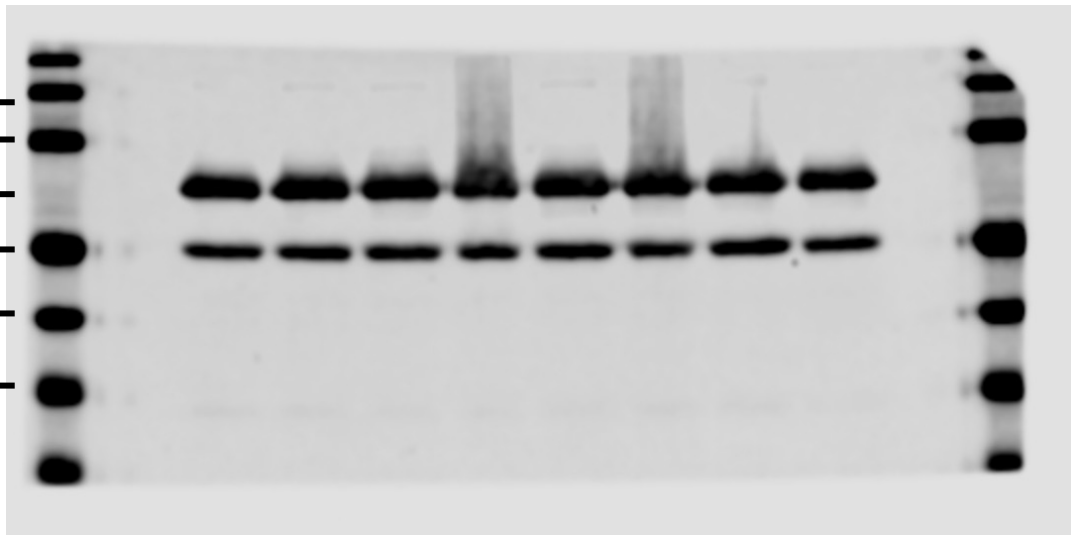

Supplement: Figure 3—source data 1. [file elife-84382-fig3-data1.zip › Figure 3 (D,E) source data/Figure 3 (D,E)-1 uncropped.pdf]

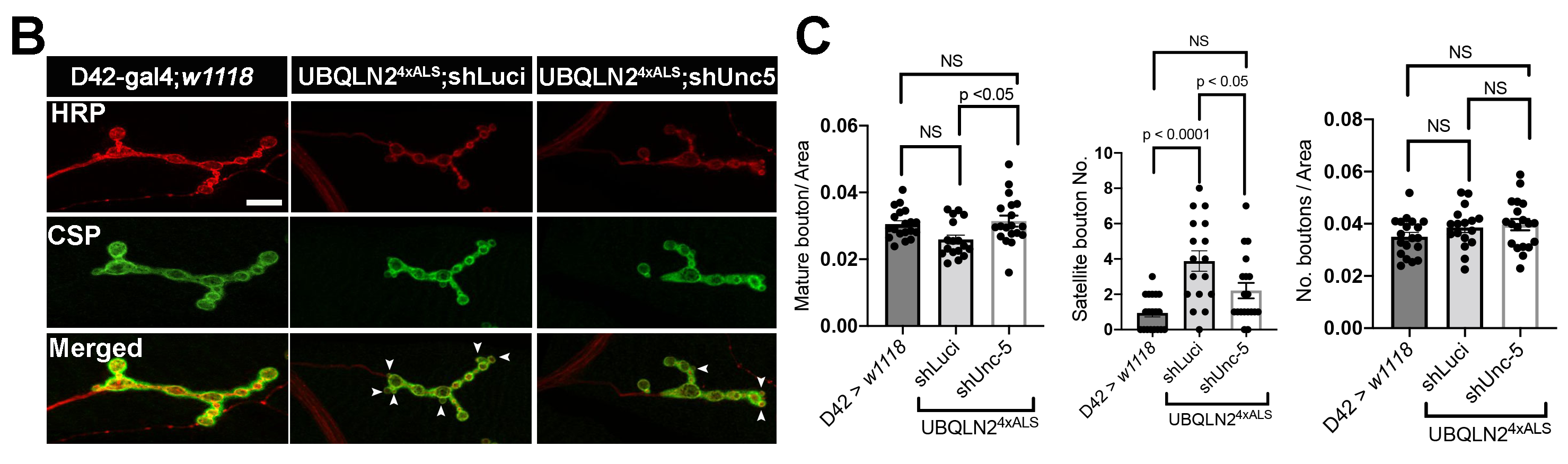

Supplement: Figure 4—source data 1. [file elife-84382-fig4-data1.zip › Figure 4 source data/Figure 4C source data/Unc5 and P4X data.tif]

Figure 8E

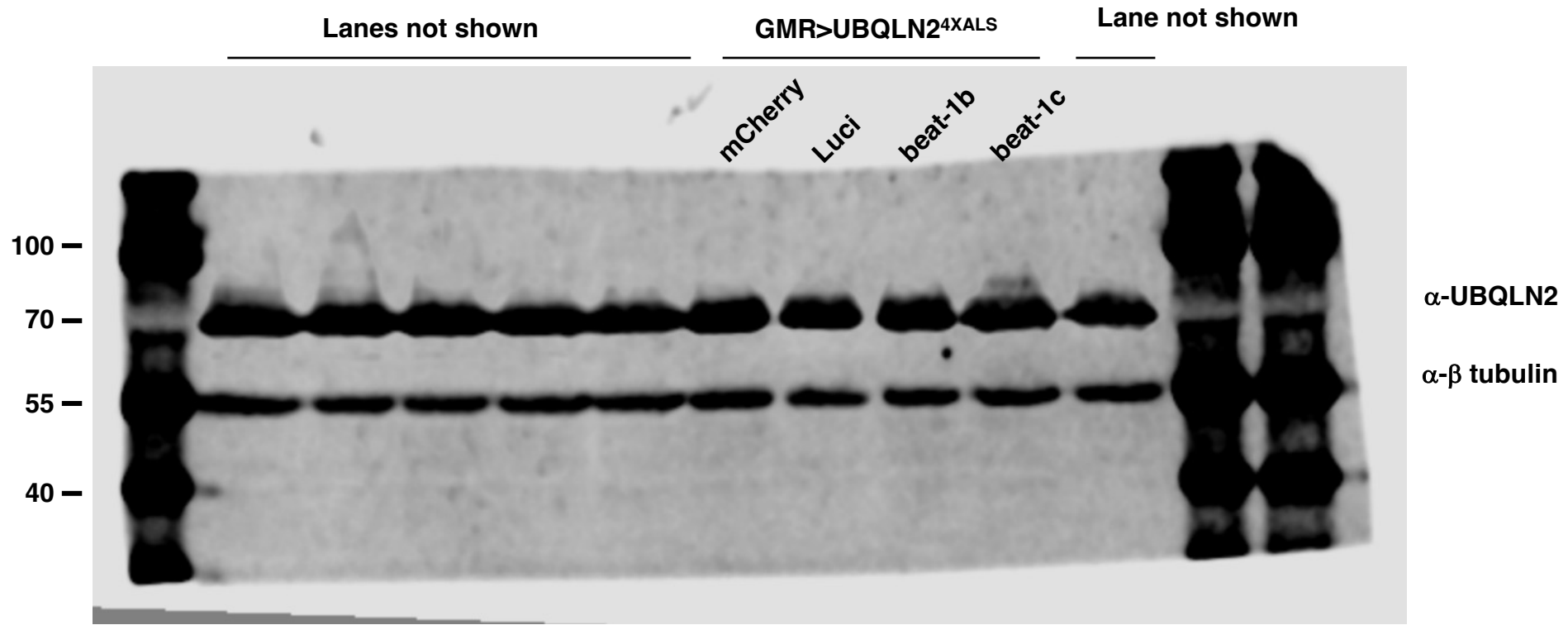

Supplement: Figure 5—source data 1. [file elife-84382-fig5-data1.zip › Figure 5 source data/Figure 5(E,F) source data/Figure 5(E,F)-3-uncropped.pdf]

# GMR>UBQLN2<sup>4XALS</sup>

mCherry

Luci

Beat-1b

Beat-1c

70

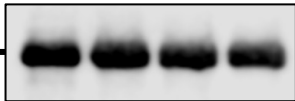

$\alpha$ -UBQLN2

55

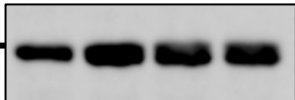

$\alpha$ - $\beta$  tubulin

Supplement: Figure 5—source data 1. [file elife-84382-fig5-data1.zip › Figure 5 source data/Figure 5(E,F) source data/Figure 5(E,F)-2.pdf]

# GMR>UBQLN2<sup>4XALS</sup>

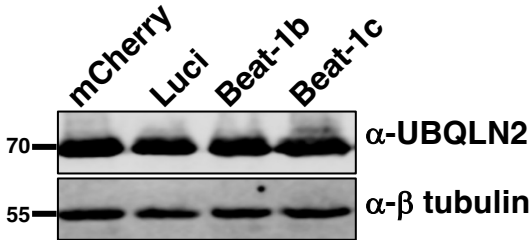

Supplement: Figure 5—source data 1. [file elife-84382-fig5-data1.zip › Figure 5 source data/Figure 5(E,F) source data/Figure 5(E,F)-3.pdf]

# GMR>UBQLN2<sup>4XALS</sup>

mCherry  
Luci  
Beat-1b  
Beat-1c

70

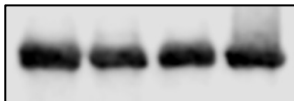

$\alpha$ -UBQLN2

55

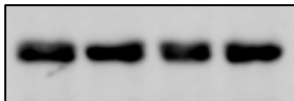

$\alpha$ - $\beta$  tubulin

Supplement: Figure 5—source data 1. [file elife-84382-fig5-data1.zip › Figure 5 source data/Figure 5(E,F) source data/Figure 5(E,F)-1.pdf]

GMR>UBQLN2<sup>4XALS</sup>

GMR>UBQLN2<sup>4XALS</sup>

mCherry  
Luci  
Beat-1b  
Beat-1c

mCherry  
Luci  
Beat-1b  
Beat-1c

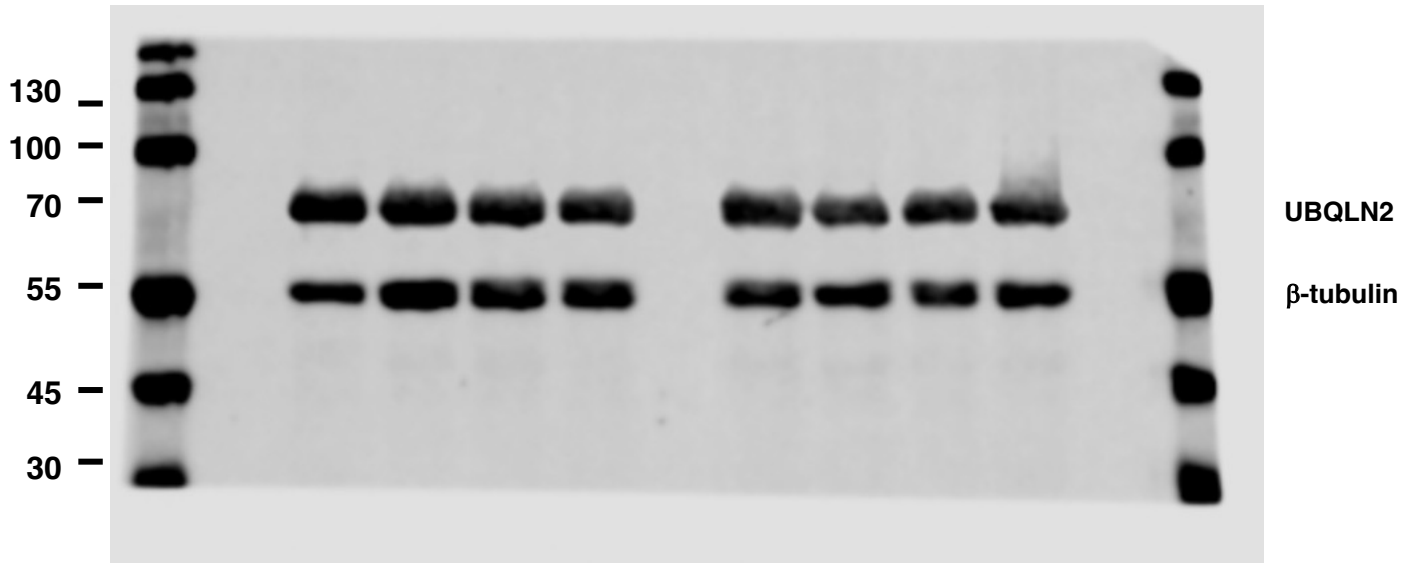

Supplement: Figure 5—source data 1. [file elife-84382-fig5-data1.zip › Figure 5 source data/Figure 5(E,F) source data/Figure 5(E,F)-1,2-uncropped.pdf]

Figure 8C

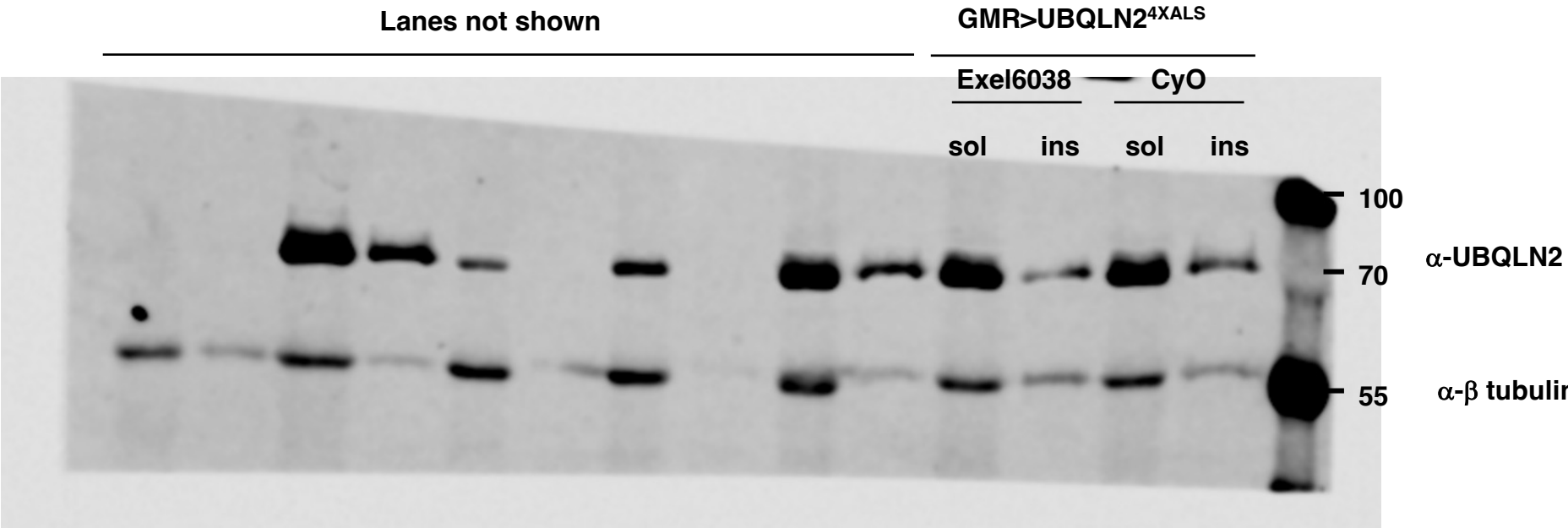

Supplement: Figure 5—source data 1. [file elife-84382-fig5-data1.zip › Figure 5 source data/Figure 5C source data/Figure 5C-uncropped.pdf]

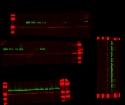

Supplement: Figure 5—source data 1. [file elife-84382-fig5-data1.zip › Figure 5 source data/Figure 5(E,F) source data/Figure 5 (E,F)-3-original/0003751_01_TH.jpg]

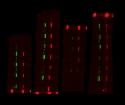

Supplement: Figure 5—source data 1. [file elife-84382-fig5-data1.zip › Figure 5 source data/Figure 5C source data/Figure 5C_original/0002693_01_TH.jpg]

100

70

55

45

35

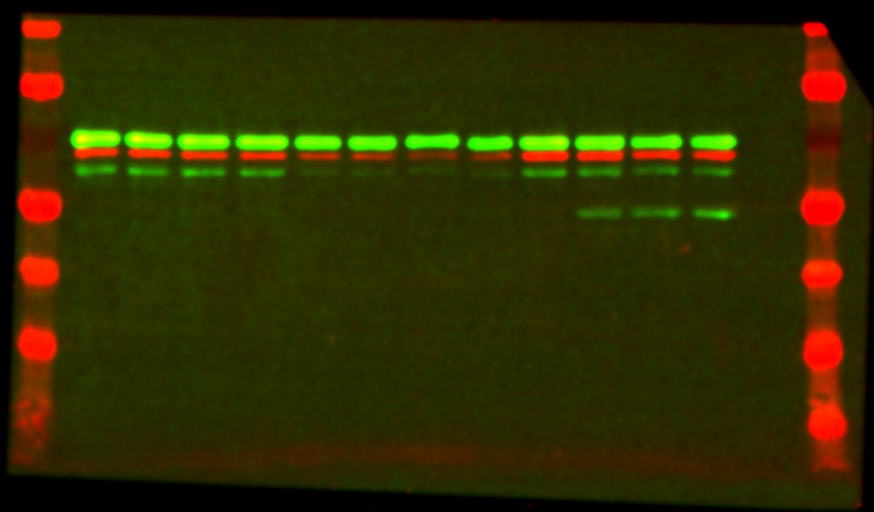

Supplement: Figure 6—source data 1. [file elife-84382-fig6-data1.zip › Figure 6 source data/Figure 6C source data/Figure 6C-2 uncropped.pdf]

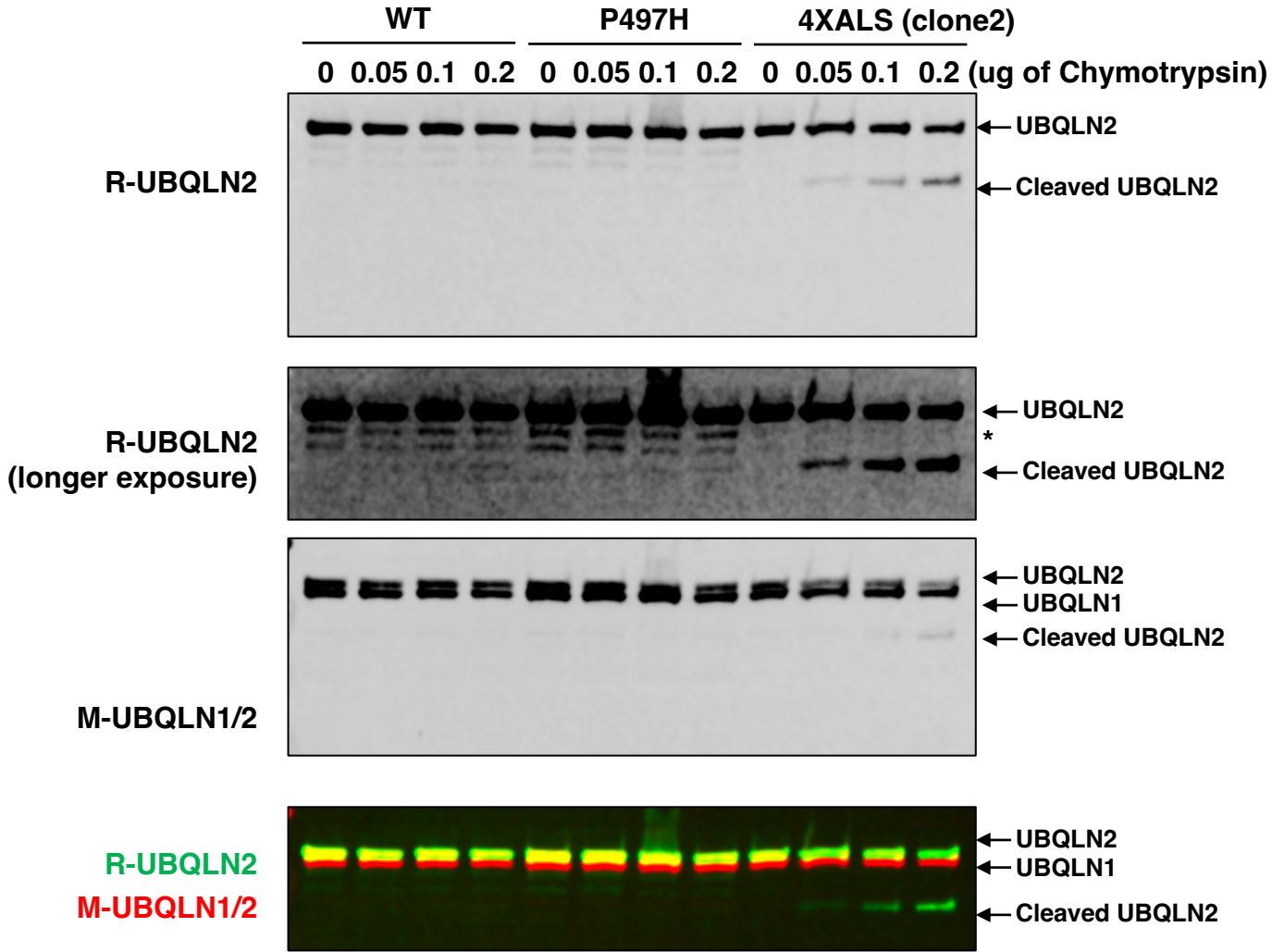

Supplement: Figure 6—source data 1. [file elife-84382-fig6-data1.zip › Figure 6 source data/Figure 6C source data/Figure 6C-1.pdf]

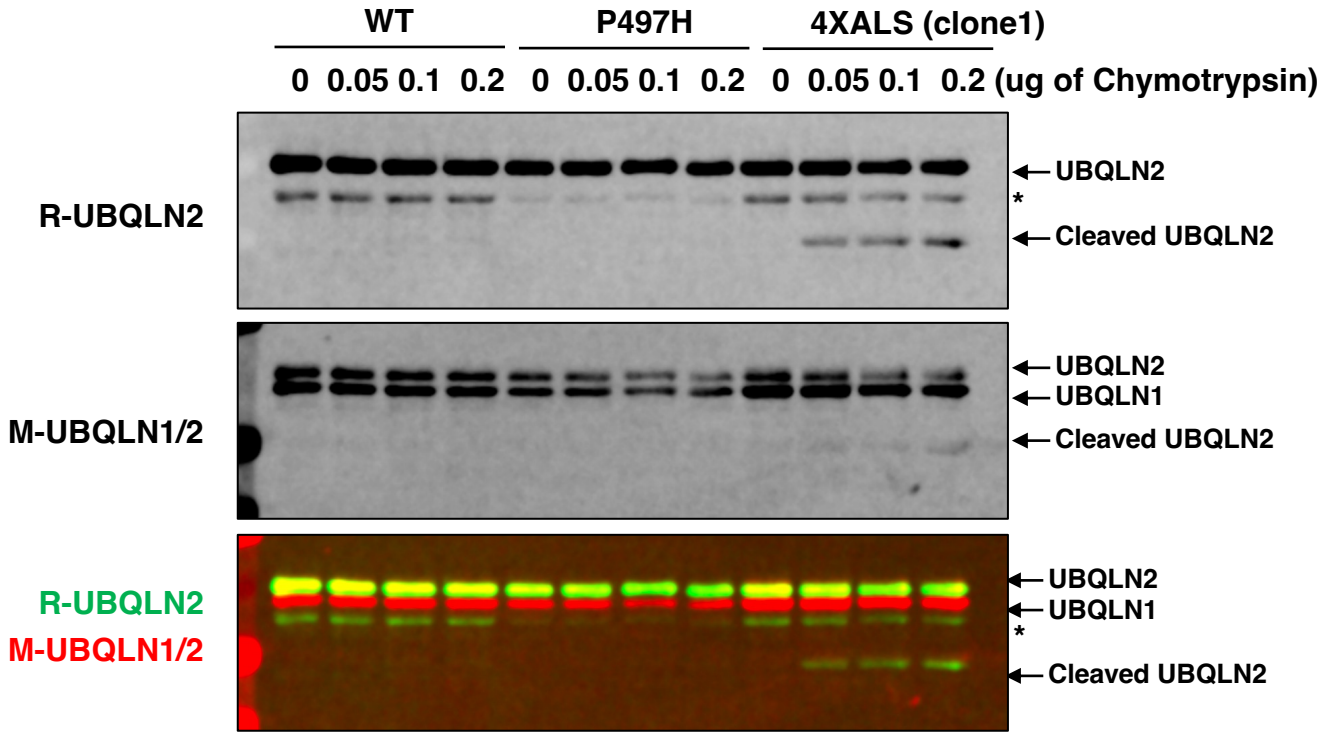

Supplement: Figure 6—source data 1. [file elife-84382-fig6-data1.zip › Figure 6 source data/Figure 6C source data/Figure 6C-2.pdf]

100

70

55

45

35

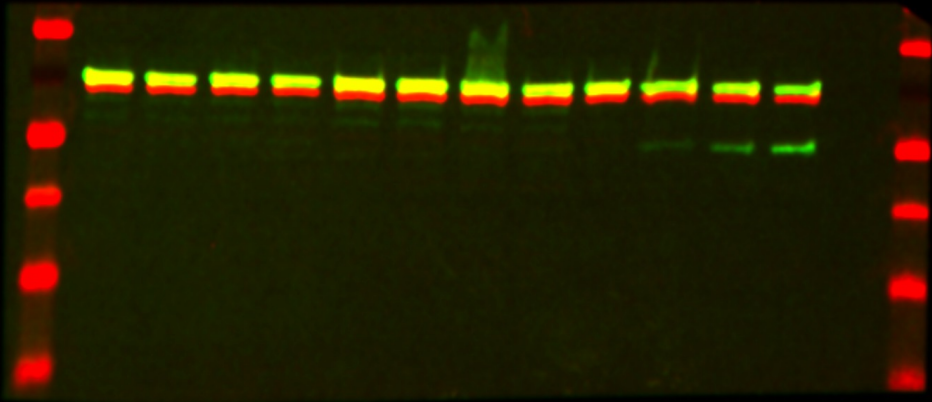

Supplement: Figure 6—source data 1. [file elife-84382-fig6-data1.zip › Figure 6 source data/Figure 6C source data/Figure 6C-1 uncropped.pdf]

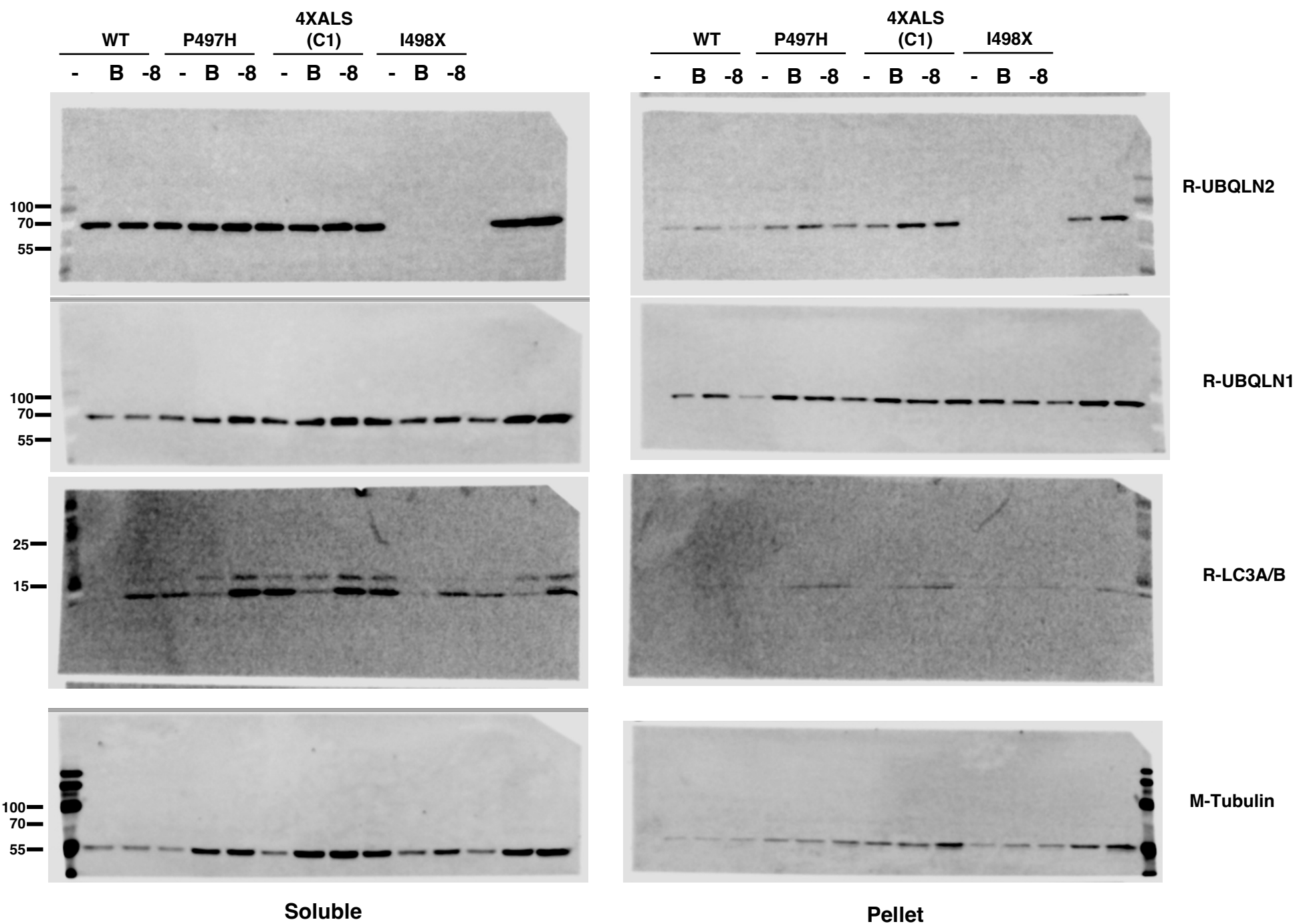

Supplement: Figure 6—source data 1. [file elife-84382-fig6-data1.zip › Figure 6 source data/Figure 6D,E source data/Figure 6D,E-1 uncropped.pdf]

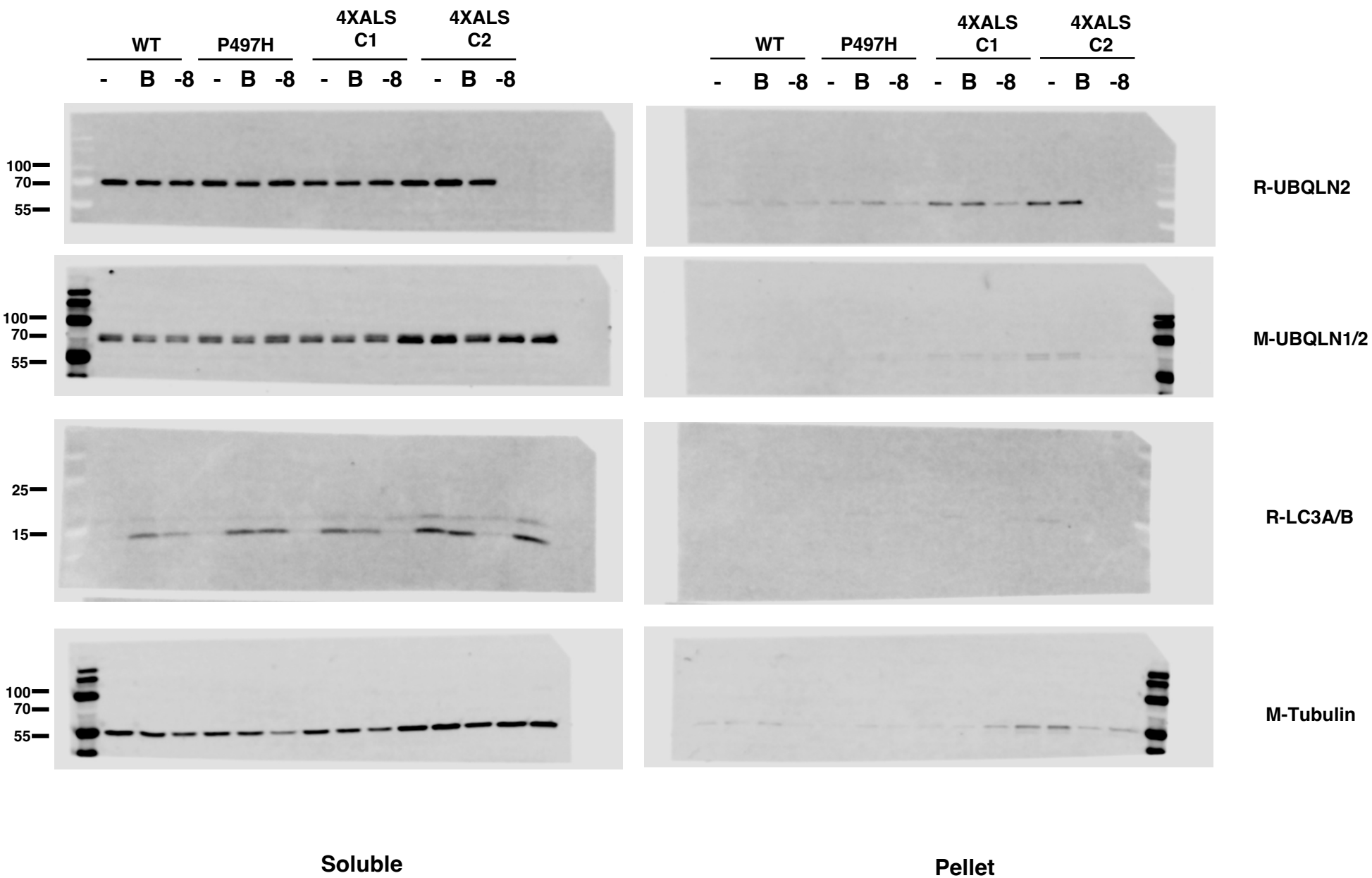

Supplement: Figure 6—source data 1. [file elife-84382-fig6-data1.zip › Figure 6 source data/Figure 6D,E source data/Figure 6D,E-2 uncropped.pdf]

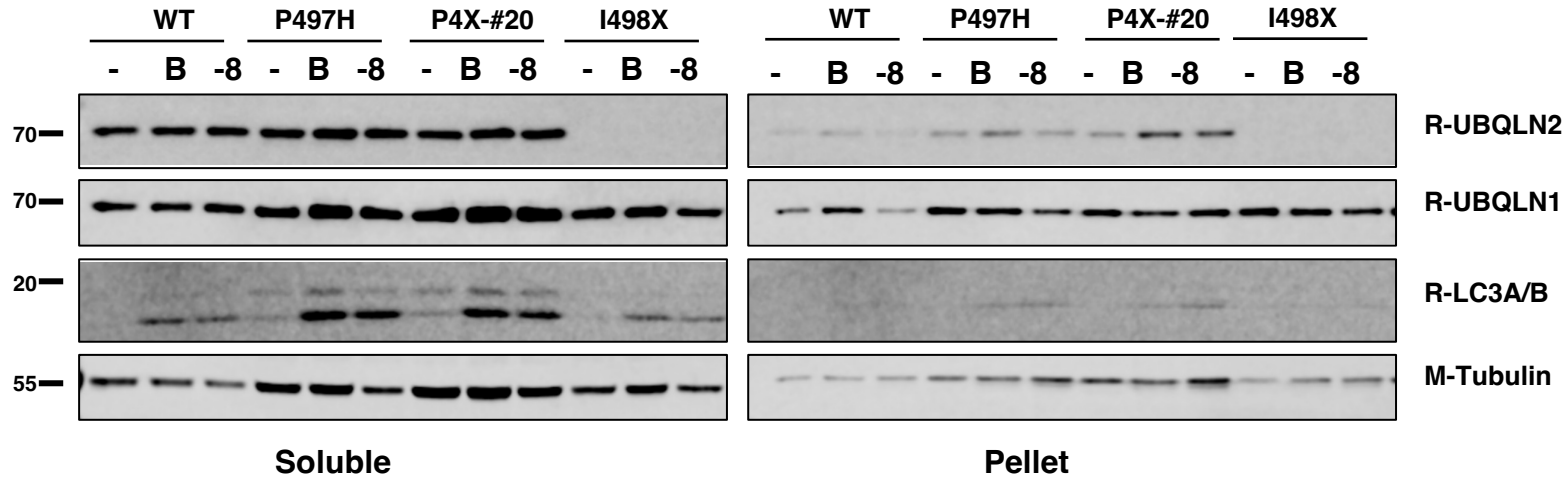

Supplement: Figure 6—source data 1. [file elife-84382-fig6-data1.zip › Figure 6 source data/Figure 6D,E source data/Figure 6D,E-1.pdf]

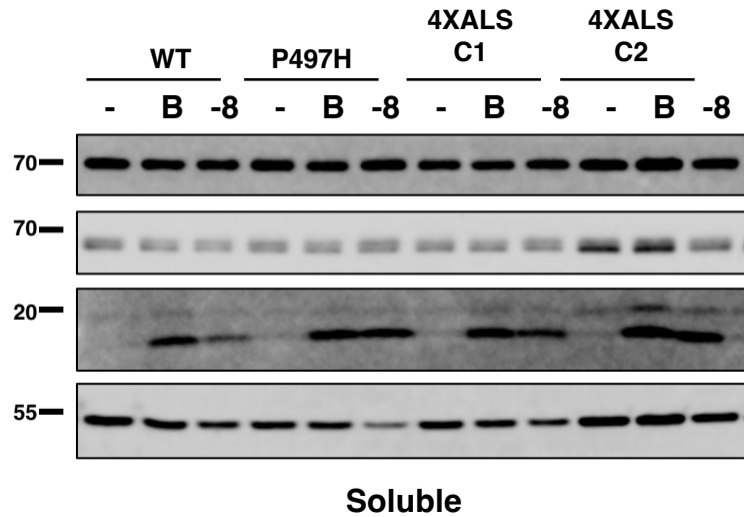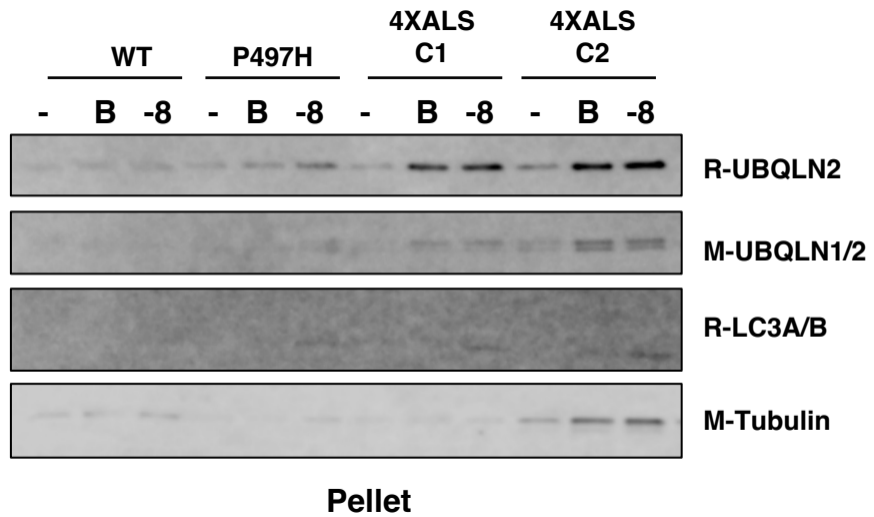

Supplement: Figure 6—source data 1. [file elife-84382-fig6-data1.zip › Figure 6 source data/Figure 6D,E source data/Figure 6D,E-2.pdf]

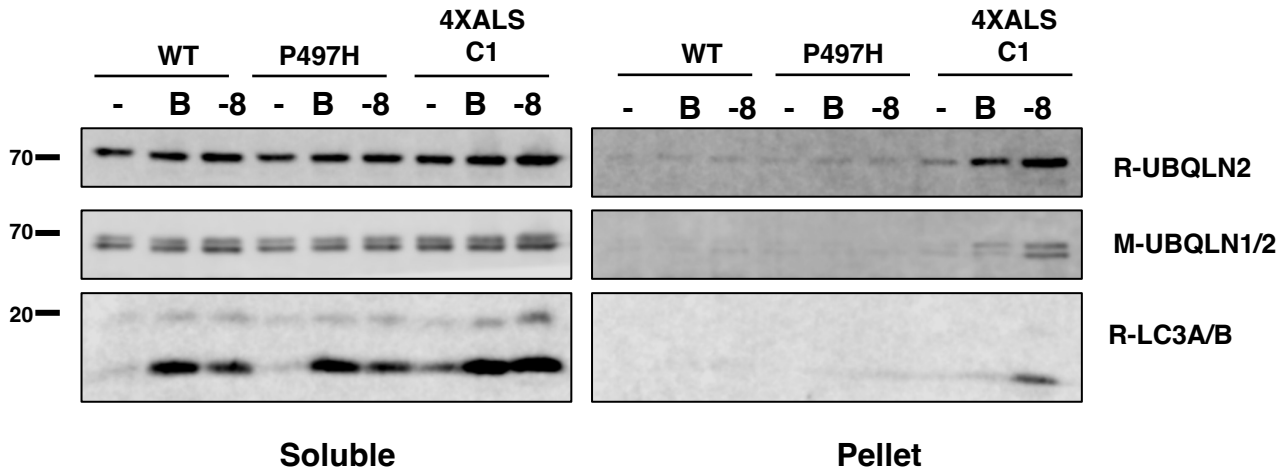

Supplement: Figure 6—source data 1. [file elife-84382-fig6-data1.zip › Figure 6 source data/Figure 6D,E source data/Figure 6D,E-3.pdf]

| WT |   |    | P497H |   |    | 4XALS C1 |   |    |
|----|---|----|-------|---|----|----------|---|----|
| -  | B | -8 | -     | B | -8 | -        | B | -8 |

| WT |   |    | P497H |   |    | 4XALS C1 |   |    |
|----|---|----|-------|---|----|----------|---|----|
| -  | B | -8 | -     | B | -8 | -        | B | -8 |

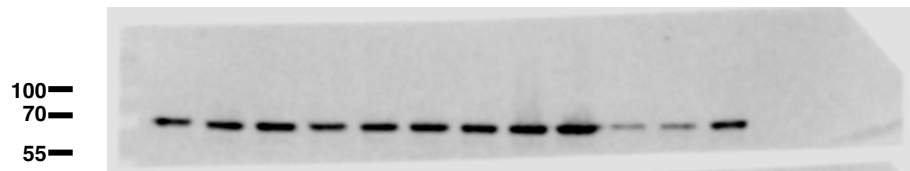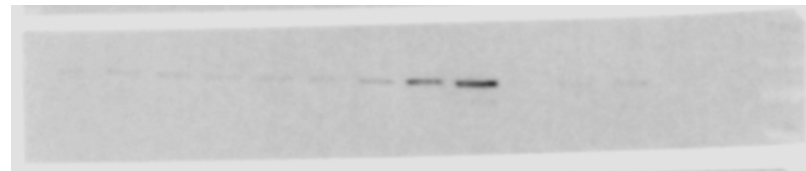

R-UBQLN2

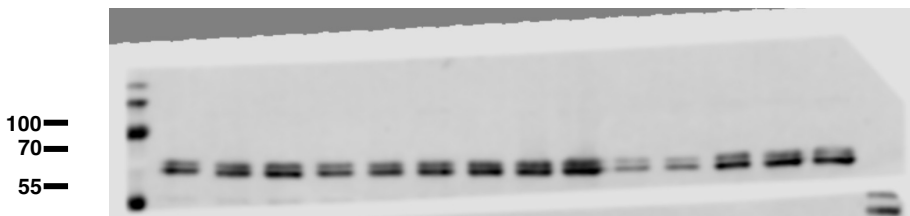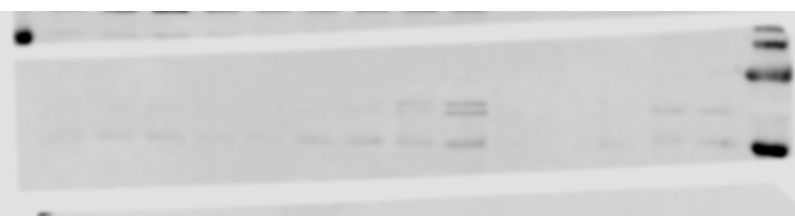

M-UBQLN1/2

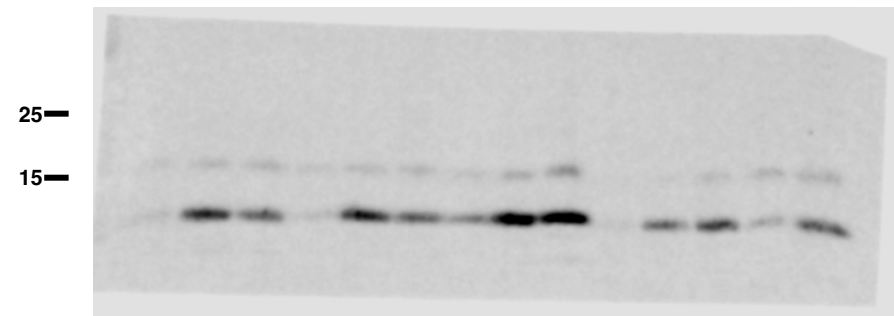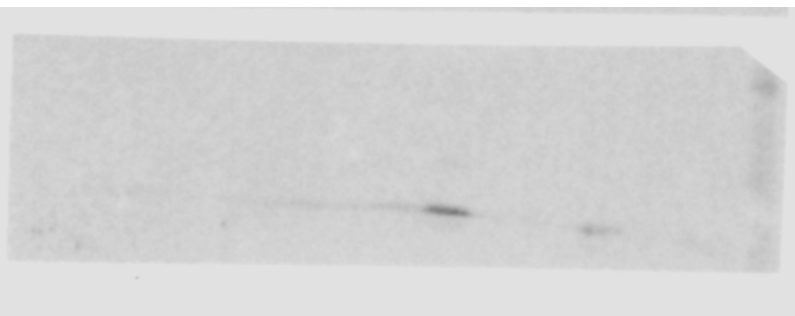

R-LC3A/B

Soluble

Pellet

Supplement: Figure 6—source data 1. [file elife-84382-fig6-data1.zip › Figure 6 source data/Figure 6D,E source data/Figure 6D,E-3 uncropped.pdf]

iPSC sol (TX-100)

iPSC pellet (TX-100)

WT P497H 2XALS 4XALS (C1) I498X

WT P497H 2XALS 4XALS (C1) I498X

70

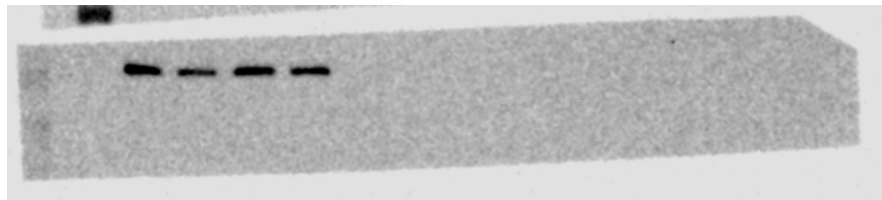

UBQLN2

100

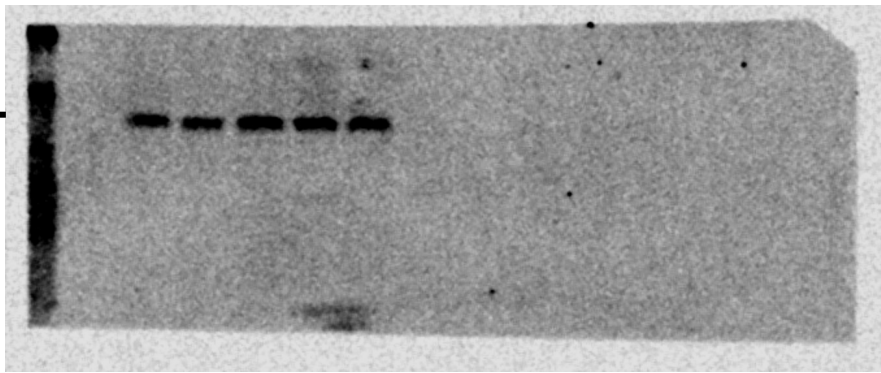

PSMD1

Supplement: Figure 6—source data 1. [file elife-84382-fig6-data1.zip › Figure 6 source data/Figure 6A source data/Figure 6A-2 uncropped.pdf]

| iPSC sol (TX-100) |       |       |       |            | iPSC pellet (TX-100) |       |       |       |            |
|-------------------|-------|-------|-------|------------|----------------------|-------|-------|-------|------------|
| WT                | P497H | 2XALS | I498X | 4XALS (C1) | WT                   | P497H | 2XALS | I498X | 4XALS (C1) |

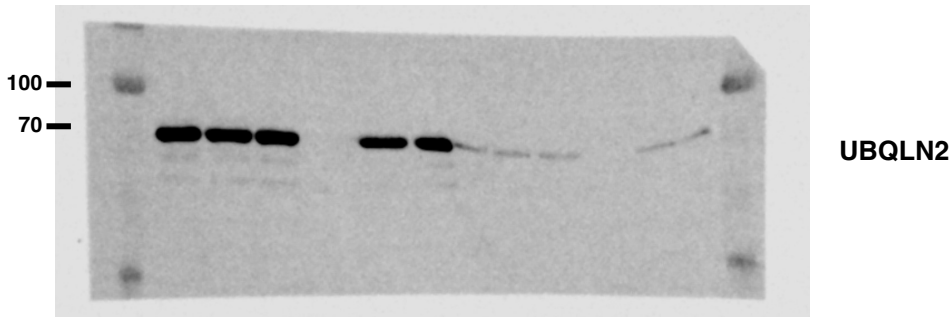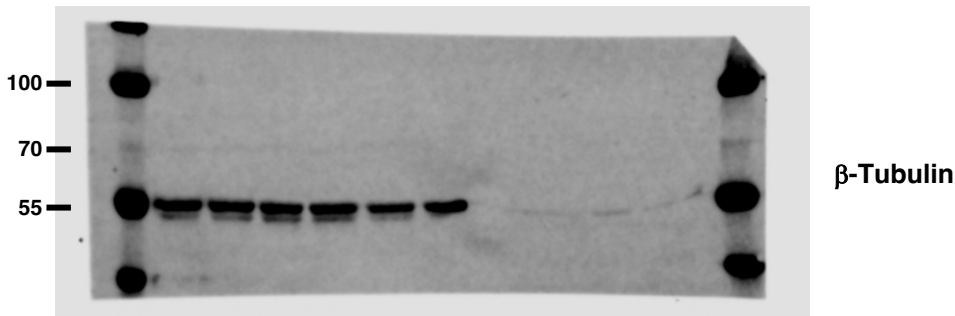

Supplement: Figure 6—source data 1. [file elife-84382-fig6-data1.zip › Figure 6 source data/Figure 6A source data/Figure 6A-1 uncropped.pdf]

iPSC sol (TX-100)

iPSC pellet (TX-100)

WT P497H 2XALS 4XALS (C1) I498X

WT P497H 2XALS 4XALS (C1) I498X

70

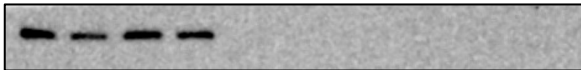

UBQLN2

100

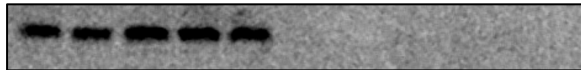

PSMD1

Supplement: Figure 6—source data 1. [file elife-84382-fig6-data1.zip › Figure 6 source data/Figure 6A source data/Figure 6A-2.pdf]

**iPSC sol (TX-100)**

**iPSC pellet (TX-100)**

**WT** **P497H** **2XALS** **4XALS** **1498X**  
**(C1)**

**WT** **P497H** **2XALS** **4XALS** **1498X**  
**(C1)**

70

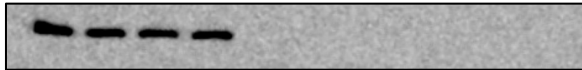

**UBQLN2**

100

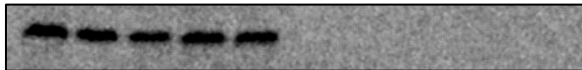

**PSMD1**

Supplement: Figure 6—source data 1. [file elife-84382-fig6-data1.zip › Figure 6 source data/Figure 6A source data/Figure 6A-3.pdf]

iPSC sol (TX-100)

iPSC pellet (TX-100)

WT P497H 2XALS 4XALS (C1) I498X

WT P497H 2XALS 4XALS (C1) I498X

70

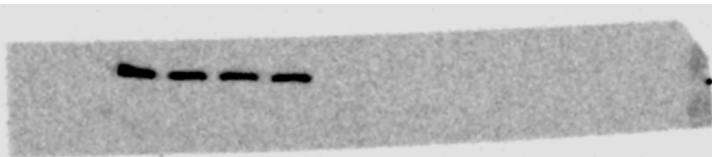

UBQLN2

100

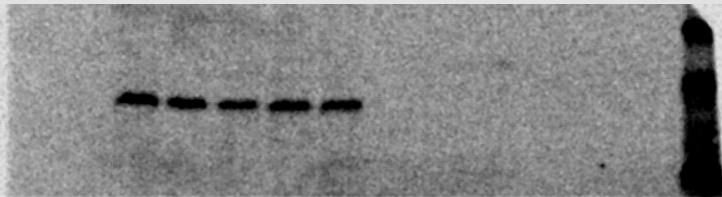

PSMD1

Supplement: Figure 6—source data 1. [file elife-84382-fig6-data1.zip › Figure 6 source data/Figure 6A source data/Figure 6A-3 uncropped.pdf]

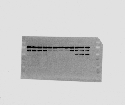

Supplement: Figure 6—source data 1. [file elife-84382-fig6-data1.zip › Figure 6 source data/Figure 6C source data/Figure 6C-1 original/0003064_01_TH.jpg]

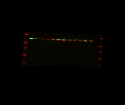

Supplement: Figure 6—source data 1. [file elife-84382-fig6-data1.zip › Figure 6 source data/Figure 6C source data/Figure 6C-2 original/0003036_01_TH.jpg]

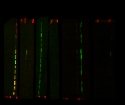

Supplement: Figure 6—source data 1. [file elife-84382-fig6-data1.zip › Figure 6 source data/Figure 6D,E source data/Figure 6D,E-3 original/0003356_01_TH.jpg]

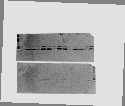

Supplement: Figure 6—source data 1. [file elife-84382-fig6-data1.zip › Figure 6 source data/Figure 6D,E source data/Figure 6D,E-1 original-3/0003326_02_TH.jpg]

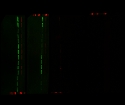

Supplement: Figure 6—source data 1. [file elife-84382-fig6-data1.zip › Figure 6 source data/Figure 6D,E source data/Figure 6D,E-1 original-2/0003323_01_TH.jpg]

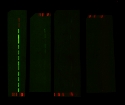

Supplement: Figure 6—source data 1. [file elife-84382-fig6-data1.zip › Figure 6 source data/Figure 6D,E source data/Figure 6D,E-2 original-1/0003334_01_TH.jpg]

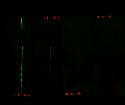

Supplement: Figure 6—source data 1. [file elife-84382-fig6-data1.zip › Figure 6 source data/Figure 6D,E source data/Figure 6D,E-1 original-1/0003322_01_TH.jpg]

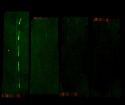

Supplement: Figure 6—source data 1. [file elife-84382-fig6-data1.zip › Figure 6 source data/Figure 6D,E source data/Figure 6D,E-2 original-3/0003337_01_TH.jpg]

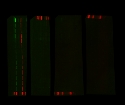

Supplement: Figure 6—source data 1. [file elife-84382-fig6-data1.zip › Figure 6 source data/Figure 6D,E source data/Figure 6D,E-2 original-2/0003335_01_TH.jpg]

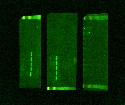

Supplement: Figure 6—source data 1. [file elife-84382-fig6-data1.zip › Figure 6 source data/Figure 6A source data/0002108_01/0002108_01_TH.jpg]

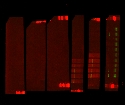

Supplement: Figure 6—source data 1. [file elife-84382-fig6-data1.zip › Figure 6 source data/Figure 6A source data/0002107_01/0002107_01_TH.jpg]

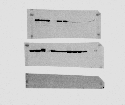

Supplement: Figure 6—source data 1. [file elife-84382-fig6-data1.zip › Figure 6 source data/Figure 6A source data/0001971_01/0001971_01_TH.jpg]
