# Supplementary material for: Axon guidance genes modulate neurotoxicity of ALS-associated UBQLN2
Source: eLife. 2023 Apr 11;12:e84382. doi: 10.7554/eLife.84382 (PMC10147378; doi:10.7554/eLife.84382)
Supplement: Figure 2—source data 1. [file elife-84382-fig2-data1.zip › Figure 2_source data 1.pptx]

## Slide 1
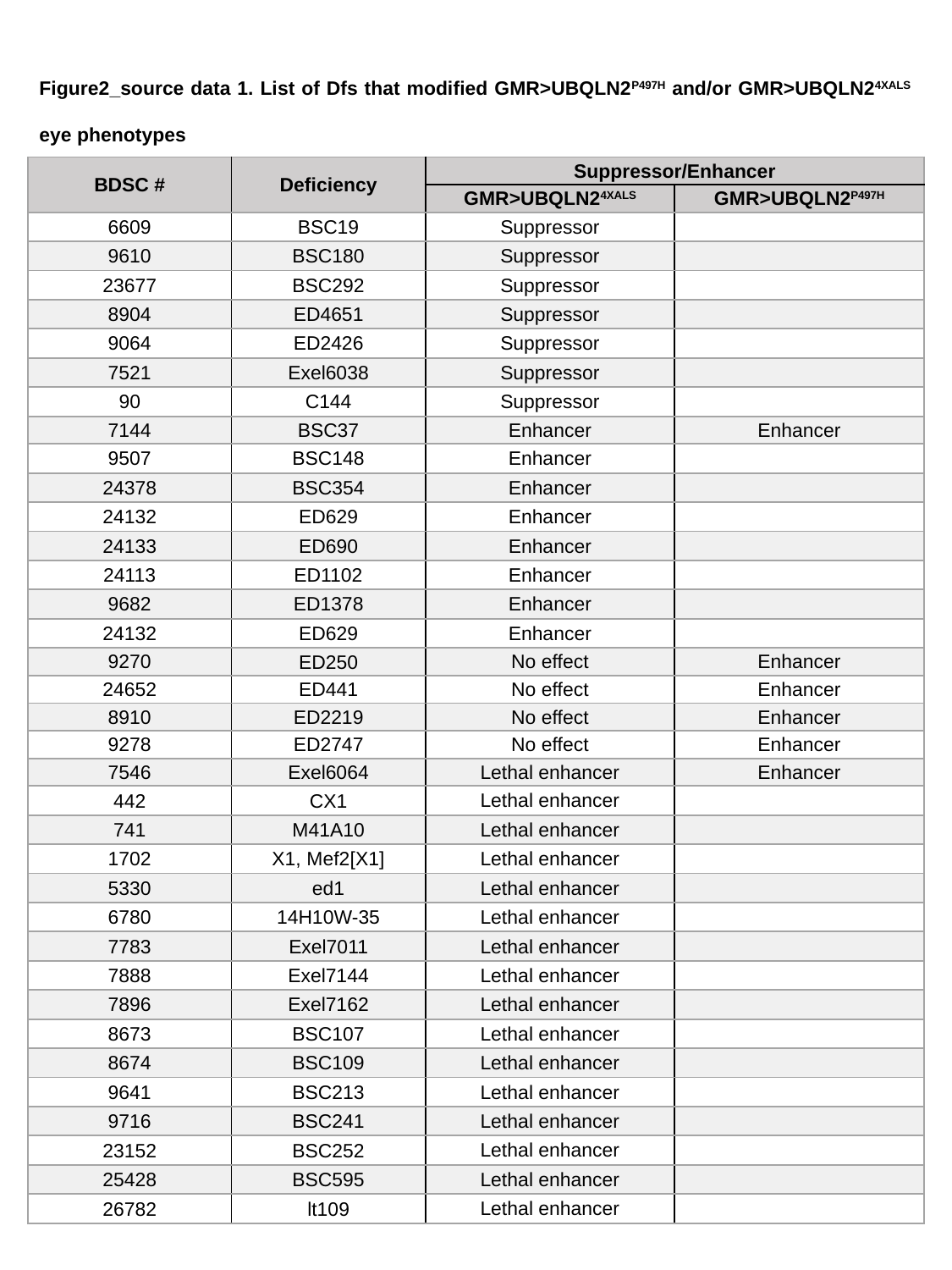

Figure2_source data 1. List of Dfs that modified GMR>UBQLN2P497H and/or GMR>UBQLN24XALS eye phenotypes
| BDSC # | Deficiency | Suppressor/Enhancer | |
| --- | --- | --- | --- |
| | | GMR>UBQLN24XALS | GMR>UBQLN2P497H |
| 6609 | BSC19 | Suppressor | |
| 9610 | BSC180 | Suppressor | |
| 23677 | BSC292 | Suppressor | |
| 8904 | ED4651 | Suppressor | |
| 9064 | ED2426 | Suppressor | |
| 7521 | Exel6038 | Suppressor | |
| 90 | C144 | Suppressor | |
| 7144 | BSC37 | Enhancer | Enhancer |
| 9507 | BSC148 | Enhancer | |
| 24378 | BSC354 | Enhancer | |
| 24132 | ED629 | Enhancer | |
| 24133 | ED690 | Enhancer | |
| 24113 | ED1102 | Enhancer | |
| 9682 | ED1378 | Enhancer | |
| 24132 | ED629 | Enhancer | |
| 9270 | ED250 | No effect | Enhancer |
| 24652 | ED441 | No effect | Enhancer |
| 8910 | ED2219 | No effect | Enhancer |
| 9278 | ED2747 | No effect | Enhancer |
| 7546 | Exel6064 | Lethal enhancer | Enhancer |
| 442 | CX1 | Lethal enhancer | |
| 741 | M41A10 | Lethal enhancer | |
| 1702 | X1, Mef2[X1] | Lethal enhancer | |
| 5330 | ed1 | Lethal enhancer | |
| 6780 | 14H10W-35 | Lethal enhancer | |
| 7783 | Exel7011 | Lethal enhancer | |
| 7888 | Exel7144 | Lethal enhancer | |
| 7896 | Exel7162 | Lethal enhancer | |
| 8673 | BSC107 | Lethal enhancer | |
| 8674 | BSC109 | Lethal enhancer | |
| 9641 | BSC213 | Lethal enhancer | |
| 9716 | BSC241 | Lethal enhancer | |
| 23152 | BSC252 | Lethal enhancer | |
| 25428 | BSC595 | Lethal enhancer | |
| 26782 | lt109 | Lethal enhancer | |
