# Supplementary material for: Axon guidance genes modulate neurotoxicity of ALS-associated UBQLN2
Source: eLife. 2023 Apr 11;12:e84382. doi: 10.7554/eLife.84382 (PMC10147378; doi:10.7554/eLife.84382)
Supplement: Figure 2—figure supplement 2—source data 1. [file elife-84382-fig2-figsupp2-data1.zip › Figure 2-Figure supplement 2 source data/Figure 2-Figure supplement 2 (C)-uncropped.pdf]

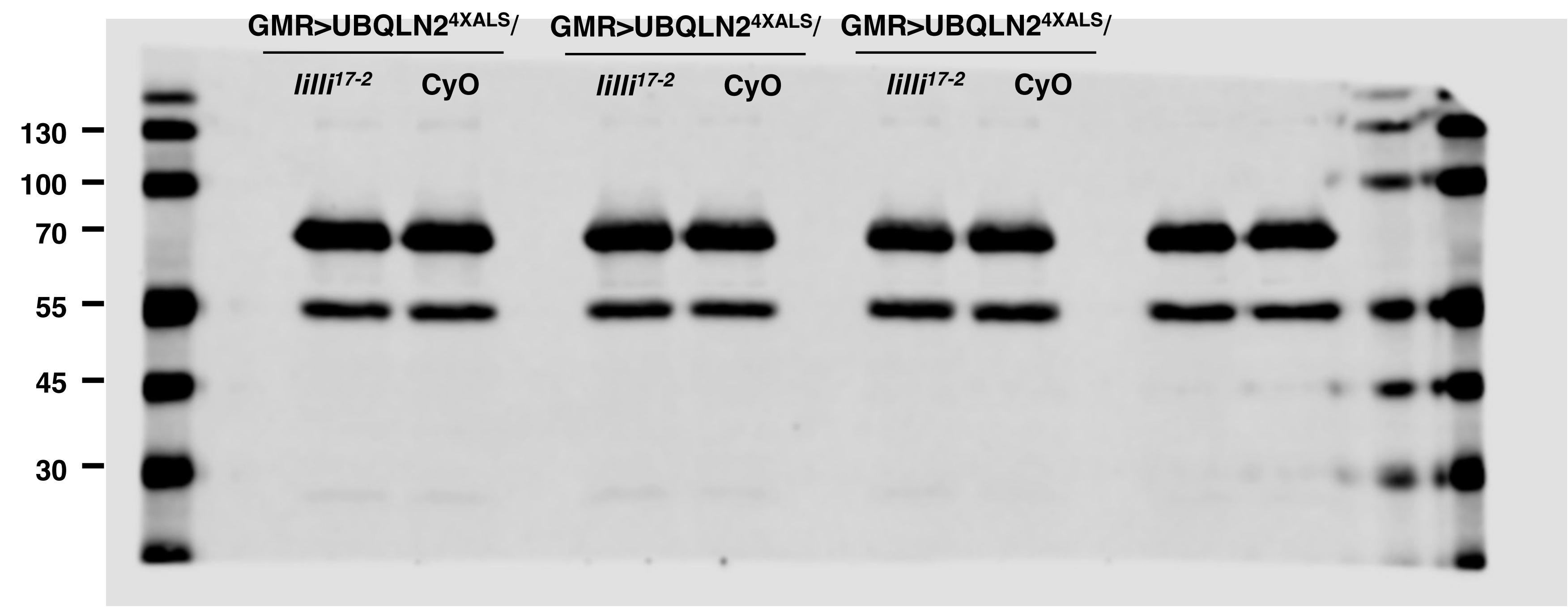
GMR>UBQLN2^{4XALS}/
GMR>UBQLN2^{4XALS}/
GMR>UBQLN2^{4XALS}/
lilli$^{17-2}$
CyO
lilli$^{17-2}$
CyO
lilli$^{17-2}$
CyO
130
100
70
55
45
30
