# Supplementary material for: Axon guidance genes modulate neurotoxicity of ALS-associated UBQLN2
Source: eLife. 2023 Apr 11;12:e84382. doi: 10.7554/eLife.84382 (PMC10147378; doi:10.7554/eLife.84382)
Supplement: Figure 6—source data 1. [file elife-84382-fig6-data1.zip › Figure 6 source data/Figure 6A source data/Figure 6A-1.pdf]

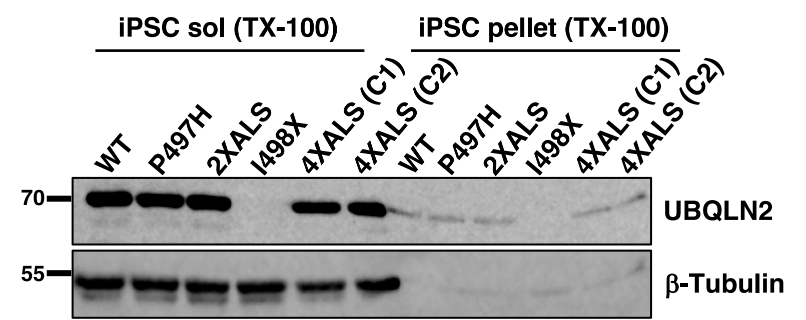
iPSC sol (TX-100)
iPSC pellet (TX-100)
WT
P497H
2XALS
I498X
4XALS (C1)
4XALS (C2)
WT
P497H
2XALS
I498X
4XALS (C1)
4XALS (C2)
70
55
UBQLN2
β-Tubulin
