# Supplementary material for: Axon guidance genes modulate neurotoxicity of ALS-associated UBQLN2
Source: eLife. 2023 Apr 11;12:e84382. doi: 10.7554/eLife.84382 (PMC10147378; doi:10.7554/eLife.84382)
Supplement: Figure 1—figure supplement 1—source data 1. [file Figure 1-Figure supplement 1-3 uncropped.pdf › Figure 1-Figure supplement 1 (A,B)/Figure 1-Figure supplement 1-3 uncropped.pdf]

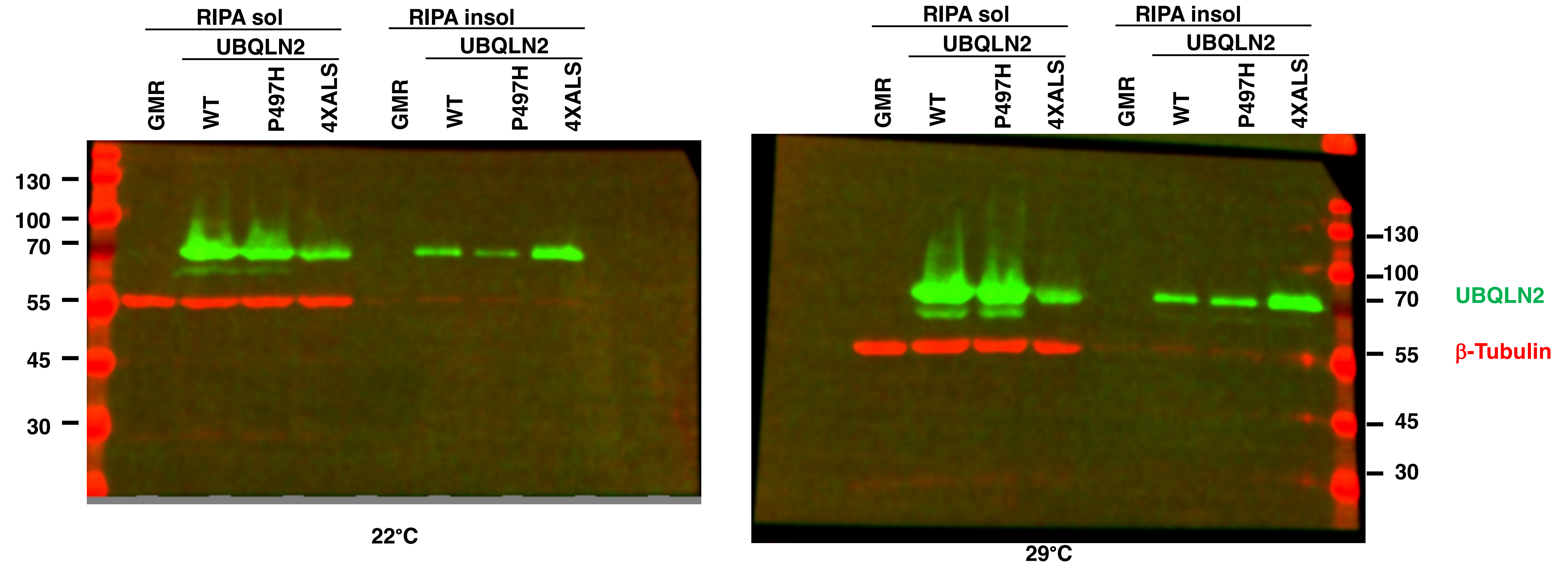
RIPA sol
UBQLN2
GMR
WT
P497H
4XALS
RIPA insol
UBQLN2
GMR
WT
P497H
4XALS
130
100
70
55
45
30
22°C
RIPA sol
UBQLN2
GMR
WT
P497H
4XALS
RIPA insol
UBQLN2
GMR
WT
P497H
4XALS
130
100
70
55
45
30
UBQLN2
β-Tubulin
29°C
